# An Inexpensive, High-Precision, Modular Spherical Treadmill Setup Optimized for *Drosophila* Experiments

**DOI:** 10.3389/fnbeh.2021.689573

**Published:** 2021-07-16

**Authors:** Frank Loesche, Michael B. Reiser

**Affiliations:** Janelia Research Campus, Howard Hughes Medical Institute, Ashburn, VA, United States

**Keywords:** *Drosophila melanogaster*, optomotor response, tethered fly, walking behavior, sensorimotor behavior, open-source, open-hardware

## Abstract

To pursue a more mechanistic understanding of the neural control of behavior, many neuroethologists study animal behavior in controlled laboratory environments. One popular approach is to measure the movements of restrained animals while presenting controlled sensory stimulation. This approach is especially powerful when applied to genetic model organisms, such as *Drosophila melanogaster*, where modern genetic tools enable unprecedented access to the nervous system for activity monitoring or targeted manipulation. While there is a long history of measuring the behavior of body- and head-fixed insects walking on an air-supported ball, the methods typically require complex setups with many custom components. Here we present a compact, simplified setup for these experiments that achieves high-performance at low cost. The simplified setup integrates existing hardware and software solutions with new component designs. We replaced expensive optomechanical and custom machined components with off-the-shelf and 3D-printed parts, and built the system around a low-cost camera that achieves 180 Hz imaging and an inexpensive tablet computer to present view-angle-corrected stimuli updated through a local network. We quantify the performance of the integrated system and characterize the visually guided behavior of flies in response to a range of visual stimuli. In this paper, we thoroughly document the improved system; the accompanying repository incorporates CAD files, parts lists, source code, and detailed instructions. We detail a complete ~$300 system, including a cold-anesthesia tethering stage, that is ideal for hands-on teaching laboratories. This represents a nearly 50-fold cost reduction as compared to a typical system used in research laboratories, yet is fully featured and yields excellent performance. We report the current state of this system, which started with a 1-day teaching lab for which we built seven parallel setups and continues toward a setup in our lab for larger-scale analysis of visual-motor behavior in flies. Because of the simplicity, compactness, and low cost of this system, we believe that high-performance measurements of tethered insect behavior should now be widely accessible and suitable for integration into many systems. This access enables broad opportunities for comparative work across labs, species, and behavioral paradigms.

## 1. Introduction

The fly *Drosophila melanogaster* is a powerful model system for research in nearly all areas of organismal biology, and has been especially central to major discoveries in the development and function of the nervous system (Bellen et al., 2010). *Drosophila* have long been champion species for a wide range of behavioral experiments that are ideally suited to a controlled lab setting (Götz, 1964; Benzer, 1967; Heisenberg and Buchner, 1977). The low cost, small size, wide availability, and ease of breeding have made flies ideal for educational and outreach settings, especially as the first or only hand-on introduction to genetics for many students (Harbottle et al., 2016). One important benefit of popularizing *Drosophila* methods for educational settings is that cutting-edge research can become directly relevant to the experience of the students. However, it is challenging to bring modern methods in animal behavior to teaching laboratories, since most setups developed for this purpose are built from custom components that are often quite expensive or difficult to obtain. Whereas, just a few years ago, specialized components required custom machining or complex procurement, the surge of “desktop manufacturing” and tools like 3D printers and laser cutters, now enables quick prototyping for low-cost fabrication. These tools support increasing interest in citizen science and STEAM education, making it practical for makers, especially those at research institutions, to assemble even complex laboratory setups. Here we describe our efforts to optimize the accessibility and cost of a complete system for preparing and experimenting on flies using the preferred method in our laboratory—precise behavioral measurements for single, body-fixed (tethered) flies presented with controlled visual stimuli (Reiser and Dickinson, 2008; Dombeck and Reiser, 2012).

Why would anyone want to build this accessible setup for measuring visually guided fly walking behavior? We think there are at least three very good reasons. First, these experiments have been central to many recent discoveries. For example, the critical role of T4 and T5 neurons as the primary source of direction selective motion vision (Bahl et al., 2013; Strother et al., 2017), and the discovery of a compass network that tracks heading in the ellipsoid body (Seelig et al., 2010), were both discovered with fly-on-ball setups. Second, the typically used setups for carrying out these experiments are quite specialized, and therefore our updated approach may be the first to bring this complicated setup within reach for many labs. And finally, the rewards of establishing such a setup are large and immediate, since this setup produces reliable measurements of robust behaviors–many of which can even be observed by eye. Consequently, fly-on-ball setups enable efficient, quantitative experiments that are ideal for exploring new stimulus regimes or replicating prior results. We believe these experiments are also ideal for teaching students about neurobiology, for an introduction into laboratory instrumentation, and for a hands-on exposure to quantitative animal behavior and the related opportunities for stimulus designs and data analysis. We hope that the accessibility and low cost of this system makes it suitable for a wide variety of research laboratories, summer courses, undergraduate, and even high-school teaching labs.

In what follows we describe the motivation and goals of the project, then detail all the components of the system, characterize the performance of the integrated setup, demonstrate its performance in measuring rather sophisticated aspects of visually guided behavior in flies, and finally estimate the cost of our systems. While we favor a modular, adaptable approach to instrumentation, we have endeavored to simplify the described system, so we mainly detail one specific setup, but throughout we describe some alternative solutions that we considered. The manuscript describes the system that we have built and used for data collection between November 2020–May 2021. We thoroughly documented the system at https://reiserlab.github.io/Component-Designs/ and will post updates on the repository while we continue making improvements to this setup.

### 1.1. Motivation and Approach

The continual improvement of many commercial technologies comes as a direct result of massive, iterative efforts, by thousands of engineers, optimizing all aspects of the design of these products (consider that smart phones are not quite 15 years old). By comparison, even the most mature instruments used for collecting laboratory data are essentially bespoke prototypes benefiting from very few “generations” of development. For that reason, many scientists prioritize designing their setups to combine high flexibility with precise control, which often requires using fairly expensive components capable of precision that far exceeds the requirements (often overestimated since never precisely specified) of any individual experiment. For the fly behavioral setup we have sought to optimize, we now benefit from several decades of methods development by many laboratories, which means we understand the requirements of this system rather well. Consequently, by eliminating unnecessary precision and flexibility, and taking advantage of desktop manufacturing tools, we could greatly simplify these setups and can now replicate them at much lower cost.

The development of our inexpensive treadmill was initially inspired by an invitation to run a hands-on training module at the *Drosophila* Neurobiology: Genes, Circuits & Behavior course at the Cold Spring Harbor Laboratory during the summer of 2019. We wanted to give each participant the hands-on experiences of anesthetizing and tethering flies and then positioning them on a treadmill to observe walking behavior, but this required many, independent setups. Therefore, when we started replicating the typical walking fly-on-ball setup we favor in our lab (Seelig et al., 2010; Strother et al., 2017), we focused on replacing the most expensive components, one-for-one, with less costly commercial parts and some 3D-printed components. At the time of the course we had converted a setup that would cost > $16,000 to replicate, to one that we built for < $500. The course was a success—we assembled seven setups and provided rigs to small groups of student who all learned to tether flies and to position them on the treadmill. This success lead us ask whether this setup was only suitable for demonstrations or could it fully replace our typical setup? In the past year we have continued to simplify and optimize the setup, achieving our goal of reproducing a “gold-standard” data set, the “optomotor” response of walking flies (Götz and Wenking, 1973; Buchner, 1976), with its well-studied dependence on the spatial and temporal properties of the visual pattern. Due to the COVID-19 pandemic we could not return to the course during the summers of 2020 or 2021, but have continued to refine the setup, so that we can now describe a complete, full-featured, low-cost implementation of both a fly preparation setup and the experimental setup. We share all component designs at https://reiserlab.github.io/Component-Designs/, a repository we plan to update continuously.

## 2. Materials and Methods

### 2.1. System Overview

**Figure 1:**
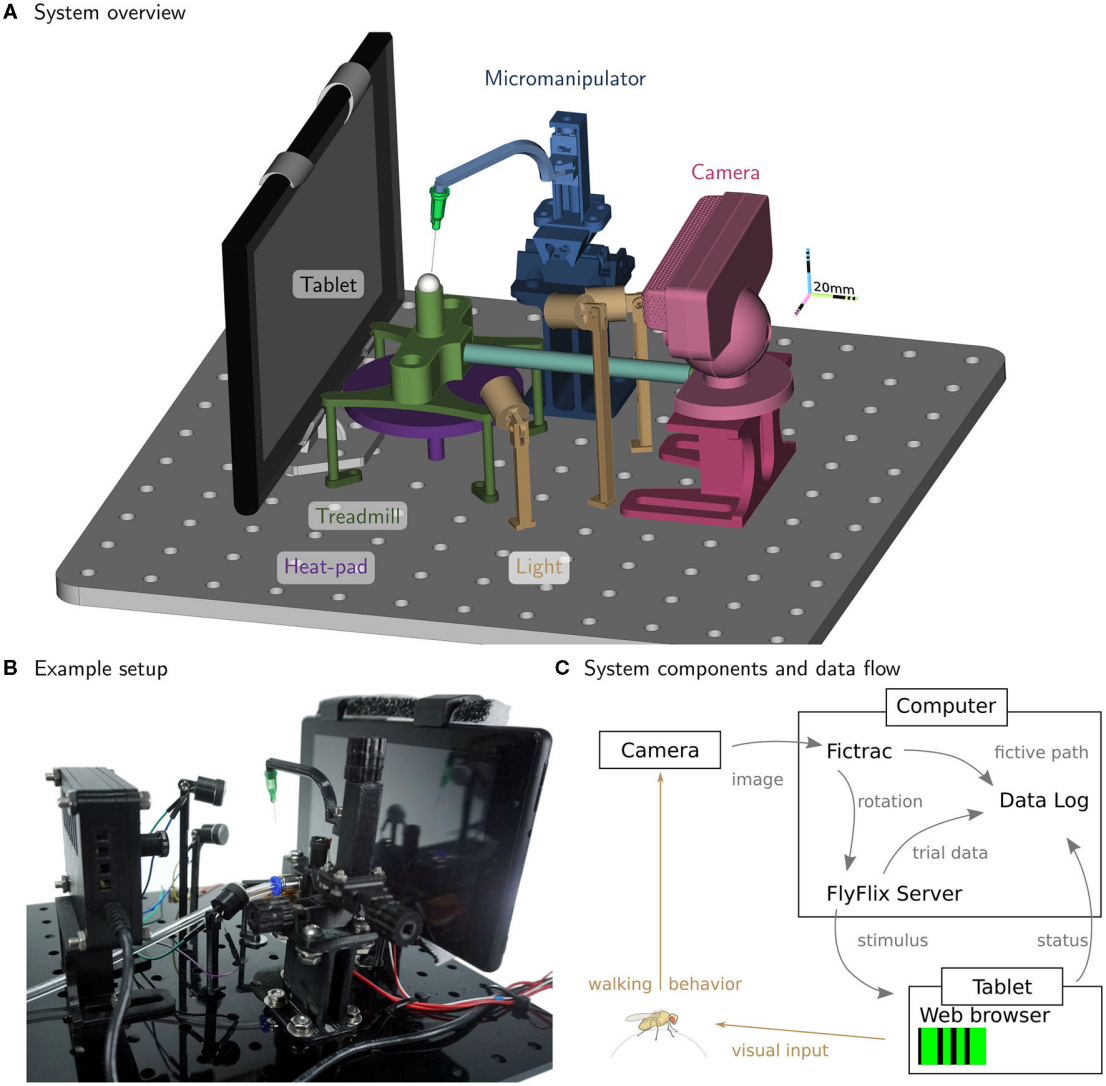
Inexpensive treadmill setup for walking fly experiments. The rendering in **(A)** highlights the major components. A fly is tethered to the thin syringe tip (light-green cone) and positioned and held in place with the micromanipulator (in blue) facing the tablet (gray) while walking on the treadmill sphere (in white). The treadmill holder (in green) floats the sphere on a steady stream of air supplied by the tubing (light blue). The camera (in red) is used to track the sphere rotations, while adjustable lights (in yellow) illuminate the sphere. The temperature near the fly is controlled from below by the heat-pad (in purple). All components are mounted on a breadboard laser-cut from an acrylic plate. **(B)** A photograph of the setup in the lab. **(C)** The flow of information between the major functional modules. For closed-loop experiments, ball rotations from FicTrac are routed to FlyFlix for on-line stimulus updates.

We detail the major components of our system for preparing (tethering) and measuring the walking behavior of flies. In Figures 1A,B, we show the components of the experimental setup. In this apparatus, a single fly is tethered to a rod that is mounted on a manipulator allowing for precise positioning of the animal along the three translational axes (all components color-coded; manipulator in blue, Figure 1A). The fly is positioned on top of an air-supported sphere, which serves as an omnidirectional treadmill (sphere holder in green). A heat-pad below the ball holder regulates the temperature near the fly (in purple); a thermistor attached to the holder provides the measurements for closed-loop control. Visual stimuli are displayed on a tablet computer (in gray) and the camera (in red) captures rotations of the ball in response to fly walking. Three LED fixtures (in yellow) illuminate the ball. Figure 1C shows signal flow for the system, including a computer that runs the software for ball tracking (FicTrac, Moore et al., 2014) as well as FlyFlix, the software we developed to generate stimuli and log responses.

**Figure 2:**
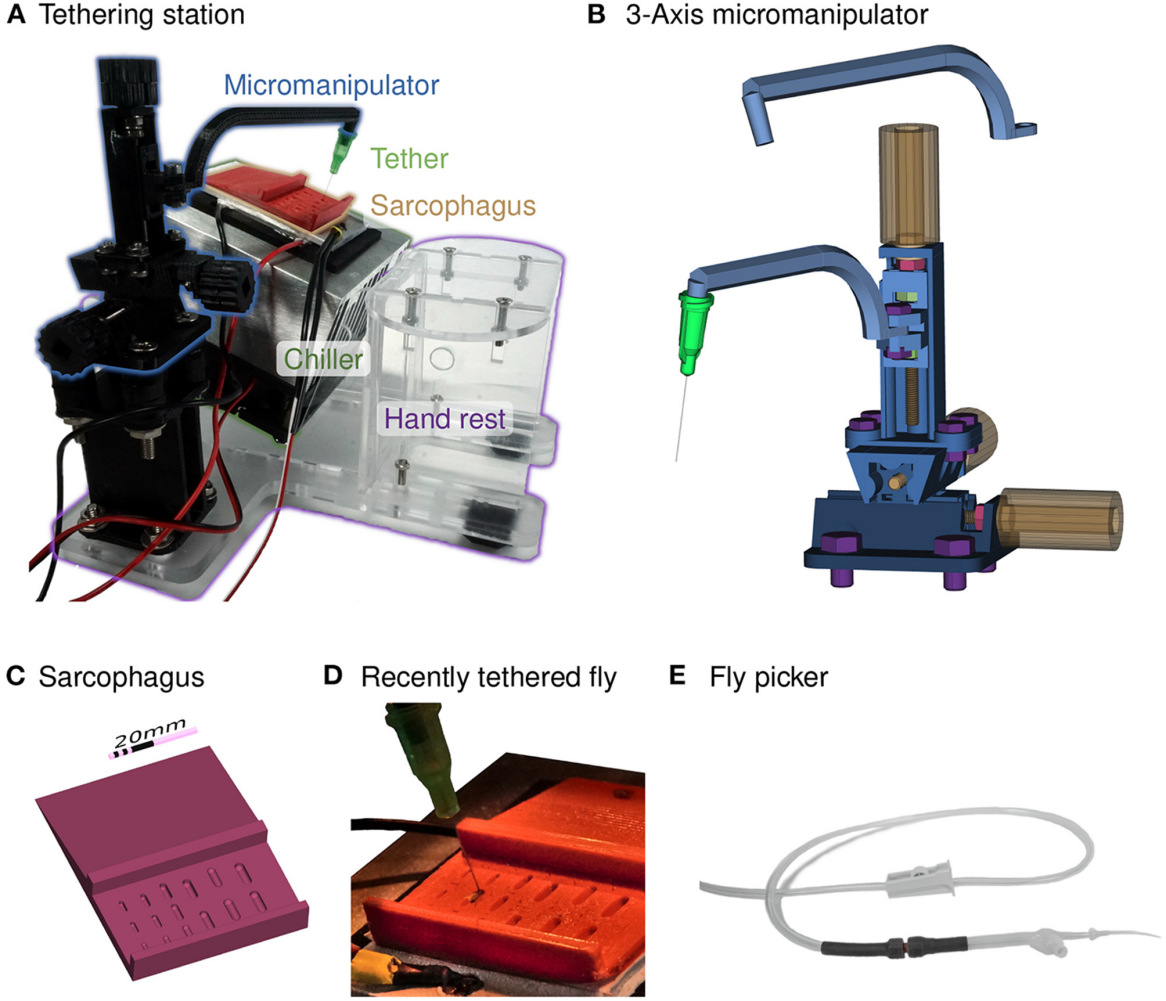
Chilled tethering station for preparing flies. **(A)** Photograph highlighting the major components of the tethering station: micromanipulator (in black), sarcophagus (in red), Peltier-based chiller with heat-sink (silver and black), and a transparent laser-cut fixture with a hand rest. This setup is typically positioned under a dissecting microscope, and a thermistor is used to measure the top surface of the chiller for closed-loop temperature control. **(B)** A compact, 3-axis micromanipulator fabricated from 3D-printed parts and simple hardware components. Two different arms are shown, one for the tethering station and the other for the experimental rig. Each axis consists of a rotating handle and screw (in yellow), a locking nut (in red) that fixes the location of the screw relative to the outer rail, and nuts within each carriage (in green), that transfer the linear motion. The device is held together and mounted using additional screws (in purple). **(C)** A rendering of the sorting and mounting plate, containing a series of indentation, each referred to as a *sarcophagus*, of different dimensions for different animal sizes. Cold-immobilized flies are sorted on the top section of the plate, and single flies are positioned in one of the cavities for gluing to the tether. **(D)** A photograph of the plate mounted on top of the chiller with a temperature sensor (yellow tape) and a fly glued to the tether (a dispensing needle). **(E)** The fly picker used to move anesthetized flies. The picker uses suction controlled by the operator's finger to pick up and deposit single flies.

Experiment on body-fixed animals have many advantages, including precise control of their sensory experience while simultaneously measuring motor output, but can be complex to implement, and often raise questions about whether the behavior is “naturalistic” (Dombeck and Reiser, 2012). Within insect behavior, there is a long history of body-fixed experiments, together with many thoughtful comparisons to the behaviors of freely moving animals. Here we describe our simplified implementation of our preferred method (Figure 2) for gluing flies to a thin rod, a process referred to as *tethering*. This process can be straightforward, but requires a specialized setup that is not widely available or particularly well-described in the literature. The goal is to mount the flies as quickly as possible and with minimal glue on a small portion of the thorax, such that this process has a minimal effect on their behavioral vigor. A good tethering strategy must enable the precision required for positioning at the small scale of the fly body, as well as the mechanical robustness required to be manipulated by human hands. Essentially, a small fly needs to be carefully glued to an object that people can routinely move from one device to another. It is nearly impossible to tether a moving fly, and so flies must first be immobilized. While there are multiple ways to anesthetize flies, and CO_2_ is commonly used, this gas affects behavior for many hours (Bartholomew et al., 2015). Instead, we favor chilling flies, which causes insects to enter a chill coma because of a transient failure of neuromuscular function, from which they rapidly recover (Findsen et al., 2014). When chilled to temperatures close to (but usually 2–4°C above) freezing, flies rapidly immobilize, but then rapidly recover upon warming (Gibert and Huey, 2001; Gibert et al., 2001). We describe the construction of the tethering station in section 2.2 and the experimental setup in section 2.3. In section 2.4, we detail how we used these components to run experiments.

### 2.2. Tethering Station

For maximal user convenience, we recommend physically separating the tethering station from the experimental setup (as in Figure 2A) and positioning it under a dissecting microscope. However, users may wish to use a single micromanipulator for both the tethering station and experimental setup (see Supplementary Figure S3D), a slightly less convenient configuration, but one that saves space and further reduces the cost.

#### 2.2.1. Magnification

In our experience, every student can learn to prepare well-glued flies for behavioral experiments with only a few sessions of practice. However, better results require learning to position flies so they are glued with approximate symmetry—in the center of the anterior notum and with minimal body rotation about the roll and yaw axes, and with the tether glued at 90° to the body long axis. This precision requires magnification. In our current setup, we position the tethering stage below a salvaged stereo microscope (Zeiss STEMI SV8). For confirmation we have tethered flies with alternative magnification methods such as a low-cost “toy” USB microscope and a magnifying glass typically used for soldering electronics, however neither is as practical as a stereo microscope. In particular, we find that the instantaneous feedback of an all-optical system is ideal for mastering the hand-eye coordination required, while the delays in the low-cost digital microscope were quite challenging to work with. We recommend a stereo microscope with a magnification of at least 10 × (although 30 × is even better) and a clearance of at least 170 mm to support the height of the tethering station.

#### 2.2.2. Tether

The tether provides the critical interface between the humans-scale and the fly-scale. We base the tethers in our laboratory on the original three-part design of Michael Dickinson (Lehmann and Dickinson, 1997). These parts are a metal connector, a ~0.1 mm diameter tungsten rod (that gets glued to the fly), and hypodermic tubing to connect the two parts. The rod and tubing are typically sold in longer units and need to be cut to length before the assembly of the three parts. A special setup is required to assemble these components reliably. Also, the tethers can be easily bent and require regular repairs and replacement. Because of the laborious assembly and other limitations of this design, we tested many alternative options better suited to the needs of a teaching course.

In our walking setup, we obtained excellent results using unmodified blunt-tip dispensing needles. Dispensing needles with Luer adapters are widely available, manufactured to tight tolerances, inexpensive, and easy to handle. We selected 34 ga needles, featuring a stainless steel tube with an outer diameter of 0.25 mm and about 12.5 mm (0.5 in) in length (for a comparison between this dispensing needle and the traditional tether design, see Supplementary Figure S5H). This is the finest needle size that is readily available from many vendors (e.g., AG-ABSS-99D0, Bstean, China). Due to this fine size, these dispensing needles are also suited for tethered flight experiments, but can be easily bent and require careful handling. From observations of students we estimate that up to 3 tethers might need replacement during a week of experiments, even though we only had to replace one during data collection for this study. We use the inner sloped cone of the Luer lock to friction mount the tethers to our setup, and have designed mount points on the arms of the Micromanipulator in the preparatory and experimental setup (Figure 2B). It is convenient to tether several flies one after the other and hold them until the start of the experiment (see Supplementary Figure S5I), for example on a strip of upward facing M4 or 8-32 screws glued to a surface. We note that the Luer adapter is keyed with a pair of plastic tabs that can be used for alignment. We only use these as a visual aid, but this feature could facilitate more automated alignment in future setups.

#### 2.2.3. Glue and Curing

To fix the tether to the fly thorax, we use resins that polymerize upon intense illumination, conveniently converting from liquid to solid within seconds. The standard glue used in our lab is KOA 300 (Kemxert, Poly-Lite, York, PA, USA), that requires UV (320–380 nm) light to cure. We typically use a commercial spot-curing lamp, such as the SpotCure-B (Kinetic Instruments Inc., Bethel, CT, USA), as they supply high intensity illumination (that cures the resin within seconds) and feature a convenient, audible timer that allows us to achieve consistent curing. Throughout the development of our setup, we tested different glue products and have confirmed that Bondic UV liquid plastic (Bondic USA, Niagara Falls, NY, USA) and Solarez Fly Tie UV Cure (Solarez Wahoo International, Vista, CA, USA), which are both widely available, work well as the tethering glue. We have a slight preference for the viscosity of the KOA 300 glue. Bondic and the Solarez thick formula appeared to be more viscous, while the Solarez flex formula (green package) is quite similar to KOA 300. A single tube of glue typically lasts over a year and the cost differences between these options are not significant. We used KOA 300 in our experiments.

For teaching lab applications, we have used inexpensive UV-LED mini flashlights (basically a CR2032 battery with a single UV-LED) to cure KOA 300. Bondic and Solarez are available in packages with battery-operated curing lights that work well for our application. In the near future, we plan to integrate automatically timed UV curing lights into the tethering setup.

#### 2.2.4. Cooling

When cooled below 4°C *Drosophila* are rapidly and reversibly immobilized (Gibert et al., 2001), which makes it convenient to align and tether the flies, and to perform further surgeries and treatments, if so desired. While flies can be chilled on a metal stage mounted on ice (or a frozen gel ice pack), a temperature-controlled thermoelectric cooler provides a more compact and precise solution. Using the Peltier effect, a powered thermoelectric cooler moves heat from one side of the device to the other. To cool one side continually below room temperature, heat must be effectively carried away from the hot side. In our lab, we use a recirculating water chiller to pump water through a liquid-cooled Peltier assembly. This is quite reliable, but is expensive, cumbersome due to the substantial tubing required, and is occasionally very messy.

For our optimized setup we use an integrated, low-cost Peltier assembly, labeled “Chiller” in Figure 2A, that is a 40 × 40 mm^2^ thermoelectric module mounted between a 40 × 60 mm^2^ aluminum plate and a 90 × 90 mm^2^ aluminum heat-sink with a fan for cooling (Adafruit Industries, New York, NY, USA). When powered with a 12 V 5A supply, the top aluminum plate reaches temperatures below 0°C while operating under typical ambient room temperatures, confirming that the device can adequately cool flies. In order to provide a consistent temperature above freezing, we implemented closed-loop temperature control using a W1209 module (multiple vendors, e.g., MOD-78, ProtoSupplies, Lake Stevens, WA, USA) that can regulate an electric load up to 10 A based on input from a 10 kΩ NTC thermistor attached to the top side of the chiller's aluminum plate (see Figure 2D). We mount the chiller at 20° toward the experimenter, shown in Figure 2A. This angle provides good airflow for cooling the module, while pushing air away from the experimenter so as not to blow flies off of the tethering station. By pitching the platform we ensure that flies will always be visible from above while being inspected, aligned, and tethered. This means that both the fly and tip of the tether are seen throughout the process. For the simplest setup, we angled the chiller by extending two screws at the corners of the fan attached to the heat-sink (see Supplementary Figures S3A,D). The integrated setup shown in Figure 2A is assembled from laser-cut acrylic sheets, and the design also includes mounting holes for the micromanipulator and a hand rest.

#### 2.2.5. Sarcophagus

To position, hold, and sometimes dissect or manipulate cold-anaesthetized flies during tethering, we typically use a movable cylindrical cavity machined from solid brass. This design is affectionately referred to as a *sarcophagus* and based on the original design of Karl Götz (Max Planck Institute for Biological Cybernetics) from the 1960s. The most important feature of the cavity is that it should be smooth and slightly larger than a fly, since sharp edges can easily injure fly legs. Beyond this detail, many aspects of the elaborate Götz design are not required for routine tethering of flies for walking experiments. For the optimized tethering stage, we tested 3D-printed sarcophagus components produced from different materials, including resin, ABS, and TPA, and found all of them working similarly well.

We made the example plate in Figures 2A,D from red ABS. 3D printing allowed us to place cavities of different sizes on a single plate, to accommodate experimenter preference for size and depth, as well as to support tethering insects of differing sizes. The inclined sorting area on the top section of the plate effectively has different temperature zone depending on the depth of material toward the Peltier element. We sanded the bottom side of the sarcophagus plate and mounted it to the aluminum plate of the chiller with thermal adhesive tape. We find that setting the Chiller to a nominal temperature of −2°C works well for our setup but may need adjustment for different setups. To maximize fly behavioral vigor, it is ideal if flies remain immobilized while on the plate, at above freezing temperatures (Gibert and Huey, 2001), but start moving within seconds once taken off the plate.

#### 2.2.6. Micromanipulator

Tethered fly experiments require precise and stable positioning at two distinct steps: when gluing the tether to the fly and for positioning the fly on the sphere. We typically use research-grade three-axis linear stages with probe-clamps (from either Thorlabs or Siskiyou) that are primarily used for microscopic manipulation and are therefore called *micromanipulators*. These essential components of a reliable setup, commercially available for >$500, are too expensive for a teaching course. We found lower-cost three-axis micromanipulators for < $100 (e.g., LD40-LM, multiple manufactures available through Aliexpress, China) that are a suitable replacement for linear stages from lab suppliers (see Supplementary Figure S4A). However, we were interested in exploring even less expensive options, and evaluated several 3D-printed alternatives, including the micromanipulator design from Open Labware (Baden et al., 2015; Chagas et al., 2017). We find this design to be quite workable, but the footprint was challenging to incorporate into our setup. Based on these explorations, we designed our own micromanipulator, optimized for simplicity and cost, and with a compact footprint.

Our three-axis micromanipulator design (Figure 2B) assembles from nine 3D-printed parts and standard screws. For each axis, an outer rail surrounds the carriage on three sides. Each rail features a screw held in place by a locking nut (red in Figure 2B). Turning each yellow knob with the attached red screw moves the corresponding green nut, and with it the carriage. The arrangement of the three axes allows translational movement in any direction by up to 20 mm. We printed the parts from ABS on a F-170 (Stratasys Ltd, Eden Prairie, MN, USA). This design requires slightly tighter tolerances than can be relied on from the printer, so we sanded the outer faces of the carriages with 200 grit sanding paper until they slide into the rail. The rails did not require post-processing. Even though this sanding can take up to 30 min, we find this advantageous as it allows us to produce a close fit across material and printers, and thus high accuracy movement, without adapting the design. We also recommend applying a plastic lubricant to the rails (e.g., Dry-Film Lube, WD-40, San Diego, CA, USA) to increase smoothness of movement. Gluing 3D-printed (or laser cut) knobs to the screw heads allows comfortable handling of the micromanipulator. The bottom rail has additional holes for securely mounting the manipulator to the baseplate. For labs with access to a 3D printer, our design of the micromanipulator costs less than $5 in materials (including nuts and screws). Ordering the parts through 3D printing services increases the cost to around $15. In addition to printing time, ~1 h of build time is required.

We designed two arms for attaching to the z-axis carriage, as shown in Figure 2B. For tethering flies we use the arm depicted above the manipulator. It is slightly longer, and holds the tether (by a friction fit) at 20° inclined from vertical, to match the slope of the heat sink. The arm shown mounted on the carriage holds the tether at a 10° angle in the opposite direction, and is used for the experimental setup. The orientation and function of these arms can be simply modified for other specific applications. While a micromanipulator assembled from 3D-printed parts is a low-cost functional substitute, it does not replicate all properties of a commercial linear stage. In particular, the plastic parts are somewhat compliant and cannot be used with heavy loads.

#### 2.2.7. Fly Picker

Single flies need to be moved and carefully positioned on the tethering platform. It is possible to do this with forceps, but we do not recommend picking up flies destined for behavioral experiments by either their legs or wings. In our laboratory we use a commercial vacuum pump and wand with a fine tip that is typically used for handling tiny electronic components during assembly. With such a device, it is possible to gently lift a fly before depositing her into the sarcophagus in nearly the ideal position for tethering. One alternative would be to fit a standard lab aspirator (or pooter) to use a fine tip. However, we find the hand-held vacuum approach to be rather convenient and so we have fashioned a version from standard components (Figure 2E). We use a plastic transfer pipette with the bulb end cut off and replaced by a tubing connector (we used Luer locks connectors, but any tight connection would work). This connection is further strengthened with heat shrink tubing. We connect the tubing to our available lab vacuum (other suction pumps or sources of negative pressure will work, Baden et al., 2015), and control the suction from the picker with a roller clamp. We cut a hole in the side of the pipette and glued in another adapter with a flat surface. When covering this stub with a finger, the suction at the tip substantially increases. Removing the finger from the stub releases the fly. Since the opening at the tip of the transfer pipette is too wide for a *Drosophila*, we added a one-way tip (F1732011 Pipetman Expert Tips EL10ST, Gilson, Middleton, WI, USA) as in Supplementary Figure S5F or a piece of thin heat shrink tubing, as in Supplementary Figure S5G. By bending a paper-clip to a desired angle and using a heat gun on the shrink tube and plastic pipette tip, we bent the tip to an angle that allowed more convenient fly pickup. A pipette tip with an inner diameter of 0.25 mm and an outer diameter of 0.65 mm allows for convenient manipulation of flies. Fly bodies are surprisingly robust, but we nevertheless recommend adjusting the pressure (via the clamp) to just above the threshold for reliably lifting flies.

### 2.3. Experimental Setup

The major components already introduced for the walking fly-on-ball setup shown in Figure 1, are described in more detail below.

#### 2.3.1. Baseplate

Many lab setups are built on solid aluminium breadboards with threaded mounting holes from specialized lab equipment manufacturers. They are very stable and can be flexibly used for many purposes. In place of these boards, we use a 300 × 300 × 10 mm^3^ acrylic board into which we cut 144 holes of 6.35 mm diameter in a 12 × 12 grid with 2.54 mm (1 in) spacing using a laser cutter. To further simplify the design, we opted not to tap threads into the holes. We position several of our components, such as the LED lamps, with a friction fit. Other parts are stably mounted with screws and nuts. We use adhesive rubber feet at the corners to lift the baseplate and add some vibration damping. This baseplate could be further simplified to the minimal size and number of mounting holes required to fit the components in the setup, but the additional holes allow for future extensions to the apparatus. This simple design is both light and stable, ideal for carrying to teaching labs and outreach events. In the accompanying repository we provide files for laser cutter, CNC-machines, or as a blueprint for hand-drilling.

#### 2.3.2. Micromanipulator

To walk with a typical gait, the fly needs to be positioned ~0.4 mm from the surface of the sphere, and aligned to the center of the ball (see Supplementary Figure S5J). We use a second, identical micromanipulator, of our own design, described in section 2.2.6, with the arm that positions a fly so they are walking at 10°, or slightly “uphill”—based on the observation that this incline appears to improve walking performance (personal communication, Shiuan-Tze Wu). The tether is friction mounted onto the arm and can be gently rotated to align the long axis of the fly toward the screen.

#### 2.3.3. Treadmill

The omnidirectional treadmill consists of a stem that holds an air-supported sphere. Our simplified, 3D-printed design for the sphere holder is a direct adaptation of an earlier design, which was custom-machined out of aluminum (Seelig et al., 2010). The original design made use of a straight inner shaft for airflow to simplify the machining process, but this limitation does not apply to 3D-printing. We implemented a more compact design where air enters via tubing with a 90° angle to the sphere-supporting air column, as shown in Figures 1A,B. In addition, we provide CAD files for alternative designs and also for different ball sizes in the accompanying repository at https://reiserlab.github.io/Component-Designs/. While, some 3D-printing methods will produce a solid, airtight part, most printers that build up parts by fusing filament in layers may result in parts that are not airtight and will allow air to escape. Rather than require specific printing methods, we achieve a quite satisfactory performance with simple post-processing. Applying acetone to the surface of parts printed from ABS seals these holes. We find that sealing only the outer surface works well, while applying solvents to the thin inner tubing could cause clogging of the air stream and might require iterations of drilling out and applying solvents again.

The flow-rate of the air needs to be controlled: if too low, the ball won't reliably float, and if too high, the ball will be much less stable (or fly off). In a previous fly-on-ball setup pressurized air regulated by a commercial mass flow controller feeds the airflow (Seelig et al., 2010). We find no loss of performance when using an inexpensive flowmeter instead, so long as it allows fine control over the appropriate range of airflow; for example, VFA-22 (Dwyer, Michigan City, IN, USA) with a maximum of 1 L min^-1^ works well. An inexpensive roller-type tube clamp can also work well. In practice, we adjust the flow rate by visually inspecting a walking fly on the ball. In place of a pressurized air supply, we have tested an aquarium-style air pump with a maximum flow rate of 1.8 L min^-1^. We find that inexpensive pumps induce some vibrations in the ball and are continuing to investigate the ideal substitute for wall-supplied pressurized air.

#### 2.3.4. Spheres

The sphere of the treadmill is the only moving part during the walking experiment and is critical for good measurements of behavior. The sphere needs to be nearly perfect in shape with a surface not too smooth, light enough to float on the air stream and be spun by the fly, but not so light that flies can pick it up, and with low rotational inertia to enable mostly unrestricted walking by flies. We have tested many alternatives, but our preferred standard sphere is still based on the method of Seelig et al. (2010), where the spheres are cut from foam with either a file or by a CNC machine (project further documented at https://wiki.janelia.org/wiki/display/flyfizz). We find that flies walk best on a sphere cut from Last-A-Foam FR-7120 (General Plastics Manufacturing Company, Tacoma, WA, USA) to a diameter of 9 mm (density of 320 kg m^-3^, sphere weighs approx. 0.12 g). For optical tracking (under near-infrared, NIR, illumination) of sphere rotations with FicTrac (Moore et al., 2014), we paint this NIR-reflective foam with BLK3.0 paint (Stuart Semple studio, Dorset, UK), which we find to be less NIR-reflective than a black permanent marker, and thus produces high contrast features. We continue to test alternative sphere materials that will be more readily available than a hand-filed foam ball. The results will be documented in the accompanying project repository.

#### 2.3.5. Sphere Tracking Camera

In tethered walking experiments the flies are fixed in space, however their intended locomotion, as if walking on an infinite virtual plane, can be estimated from the rotation of the sphere they are turning. Several methods have been developed for measuring relative rotations of the ball, for example through optical mice sensors or via optical flow calculated with camera-based systems (Lott et al., 2007; Seelig et al., 2010; Vishniakou et al., 2019). Under ideal circumstances, these relative measurements can be calibrated for excellent accuracy, but these systems can be quite sensitive to the uniformity of the lighting and focus of the sensors, etc. By estimating the absolute position of the sphere in every frame, the tracking software FicTrac (Moore et al., 2014) is an exciting alternative approach that offers several advantages.

Fictrac maps individual camera frames of a patterned ball to a previously constructed template of the sphere's pattern to estimate the instantaneous rotation of the sphere. From the frame-by-frame estimates of the sphere's orientation, FicTrac reconstructs the animal's virtual trajectory. The software works best with sharp edges and high contrast, so Moore et al. (2014) suggest to avoid motion blur by imaging with high frame rates and short exposures. FicTrac supports industrial cameras from Flir and Basler, as well as images through OpenCV, a library for real-time machine vision with extensive support for a variety of image sources.

As FicTrac operates on high-contrast, grayscale images, downsampled to a resolution of 60 × 60 px, we realize that the ideal low-cost camera should support high frame rates at low-resolution—a combination of requirements that are nearly the opposite of most inexpensive camera sensors. We found that the PlayStation Eye camera (Sony Entertainment Corp.), developed as an input controller for action games, is an excellent solution. Using open-source drivers for the low-latency integrated video processor, we obtain access to a stable video stream of 187 fps at a resolution of 320 × 240 px. The camera's sensor OV7720 (OmniVision Technologies) was developed for low-light operations, and we found the sensor is sensitive to NIR illumination once we removed the filter attached to the lens housing. To effectively use this camera, we modified the body for easier mounting and to accept S-mount lenses, as shown in Supplementary Figures S1, S2. This modification takes between 30 and 60 min. For reliable imaging of the sphere at a working distance of 10.5 cm we mount a macro lens with 25 mm focal length and a fixed aperture (see Figure 1B and Supplementary Figures S1, S2).

The PS Eye camera is our preferred high-performance and low-cost solution. Since it is mass-produced as a toy, the camera is available from different vendors and secondary markets for around $5 to $20. The modularity of our setup and FicTrac's support for many cameras through OpenCV enables other cameras with similar properties to work. To ensure that our system works reliably on readily available PCs, we ran all tests and collected all data on an older model, multi-core x86-64 system with a maximum frequency of 3 GHz and a hard disk drive running Lubuntu 20.4 LTS. This PC was powerful enough to run two FicTrac instances as well as FlyFlix, the software we developed for stimulus presentation, experiment control, and data logging in parallel. We expect that most PCs will be able to run these experiments.

#### 2.3.6. Lighting

An important consideration for measuring visually guided behaviors is to use illumination that minimally interferes with the animal's vision. The most practical solution is to use NIR illumination since fly vision is insensitive to these longer wavelengths (Sharkey et al., 2020), but most camera sensors measure it well. As we operate our camera at high frame rates and with a fixed aperture lens, intense illumination is essential for reliable sphere tracking, yet the light cannot be so intense that it saturates regions of the image (due to the limited dynamic range of any camera).

To achieve strong, but diffuse, NIR illumination, we use three generic 840 nm LEDs placed between the camera and the treadmill, pointing toward the sphere. We designed compact 3D-printed housings that allow flexible positioning of the light sources at the top of posts that are friction fit into the holes on the baseboard, as shown in Figures 1A,B. We used pieces of a plastic bag as a diffuser in front of each lamp, attached with heat shrink tubing. For our setup, we used a 5 V power supply together with a 470 Ω current-limiting resistor. With these lamps in place we adjust the lights until we obtain images of the sphere that are bright, yet evenly lit. In our standard setup (our display set to 24% brightness), we do not need to place a visible light blocking filter on the camera, although this could improve robust ball tracking with other displays.

#### 2.3.7. Heat-Pad for Temperature Control

We typically run experiments in rooms that are climate-controlled for the comfort of humans, yet these conditions are often not ideal for flies. Walking experiments can often be conveniently made more efficient by running them at increased temperature, since flies walk more often and faster at elevated temperatures (Soto-Padilla et al., 2018) and also for using temperature-dependent genetic reagents such as Shi^ts1^ or TrpA1 (Owald et al., 2015). In our experience, flies walking at warm temperatures, below 35°C, which is considered noxious (Huey et al., 1992), engage in the same walking behaviors that flies do at room temperatures, but do so with much greater consistency, as they are less likely to pause and groom. To inexpensively support warming the fly, we installed a resistive heat-pad underneath the sphere holder, controlled by a second W1209 temperature controller (also see section 2.2.4). We attached a thermistor to the sphere holder as close to the animal as possible. The actual temperature at the animal position might be slightly different (most likely lower and should be verified if critical), and we consequently refer to the W1209 setting as the *target temperature*. For the experiments detailed below, we use a target temperature of 32°C. Users of this setup could readily modify this temperature setpoint depending on their experimental requirements, or may wish to omit this temperature control subsystem.

#### 2.3.8. Display

A surprisingly wide range of visual stimulus delivery strategies have been used for insect behavioral neuroscience: from motor operated moving objects like patterned drums, to projectors and computer monitors, to custom-made LED displays (Palermo and Theobald, 2019; Kaushik et al., 2020; Kócsi et al., 2020). In our lab, we typically use custom-made, modular LED displays configured as cylinders around the animals, to deliver stimuli with excellent temporal precision (Reiser and Dickinson, 2008 and future developments documented at https:/reiserlab.github.io/Modular-LED-Display/). We have not yet succeeded at producing an inexpensive, widely available display using LEDs, and so we explored other options.

**Figure 3:**
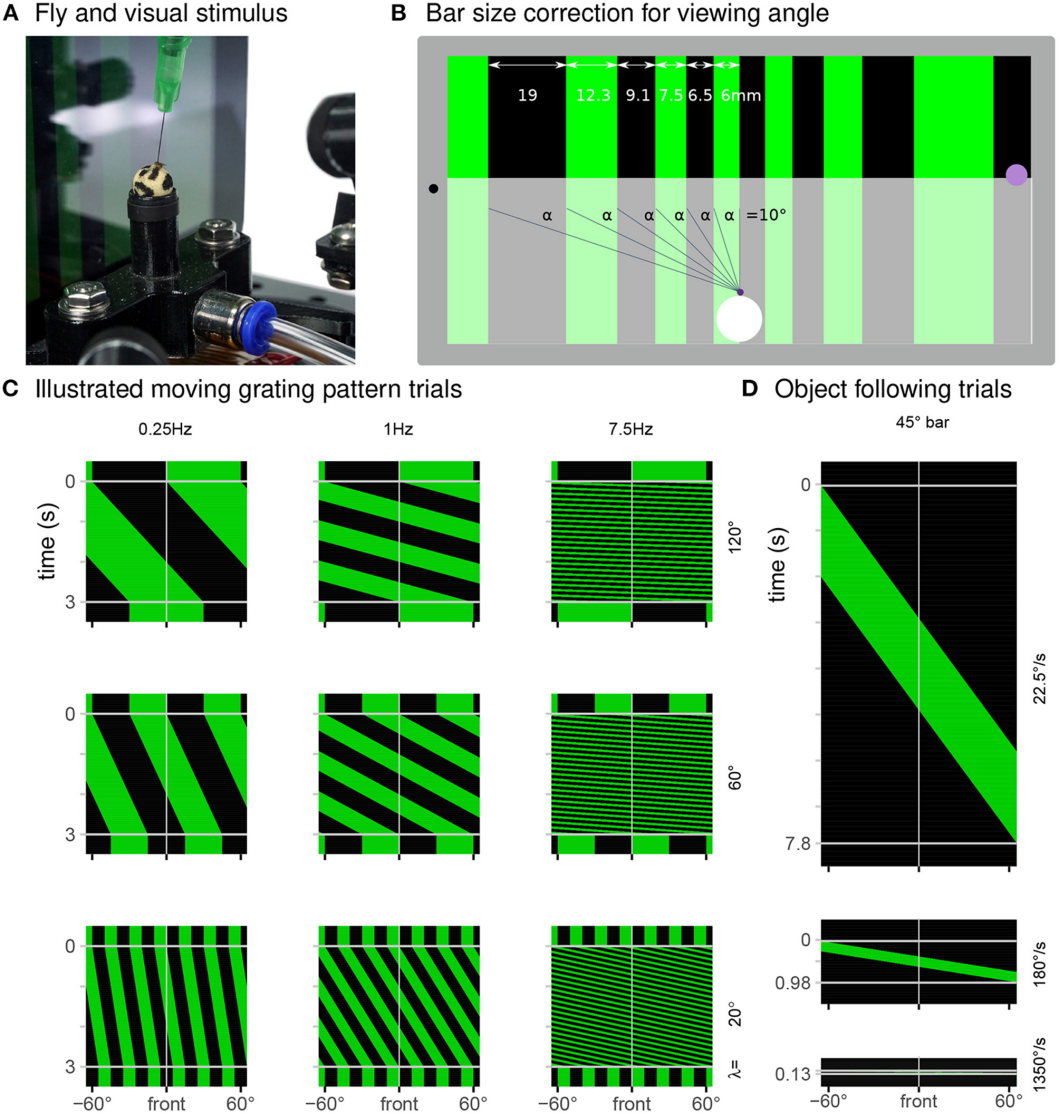
Display used to present a range of visual stimuli. **(A)** In our typical experiment, a tethered fly walks on an air-supported foam sphere, while facing a tablet computer that displays a moving grating pattern. **(B)** The FlyFlix software renders a virtual scene that simulates a cylindrical display onto the tablet's flat screen. The azimuthal span of each bar within a grating pattern is scaled to correct for the viewing angle—even though the dark bar on the left is ~3 × as wide as the bright one in the center, they both span 10° from the perspective of the fly positioned 35 mm in front of the display. The purple spot on the right marks the location where light measurements reported in Figures 4A–C were made. **(C)** Space-time representations of the display during trials showing a moving grating pattern (spatial axis displayed right-to-left, time axis is top-to-bottom). Each row of these images represents one horizontal slice through the displayed pattern, at the indicated point in time. For these 3 s trials showing moving patterns with different spatial periods and temporal frequencies, clock-wise motion appears as space-time tilts that go down and to the left. **(D)** Representation of displayed screen content during object following conditions for clockwise movement with the indicated speeds.

For the inexpensive treadmill setup, we used a widely available tablet computer with an in-plane switching (IPS) liquid-crystal display (LCD), an Amazon Fire 7 with a nominal screen size of 7 in Figure 3A. We connected the tablet to a USB power supply and to a local Wi-Fi network during all experiments and displayed visual patterns through a web browser. To allow replication across devices, we used Mozilla Firefox instead of the pre-installed browser. We installed the most recent versions of Firefox and kept the Android 9 based Fire OS updated with the latest release (most recently Firefox 86.1.x and Fire OS-7.3.x). We manually set the display brightness to 24%. IPS displays are known for their relatively wide “viewing angle,” but from the position of the fly 35 mm in front of the center, there will be an intensity gradient depending on the pixel position. For the patterns we display, this effect partially reduced since we compensate for the view-angle by increasing the physical width, and therefore the brightness, of bars closer to the edge of the screen (Figure 3B).

**Figure 4:**
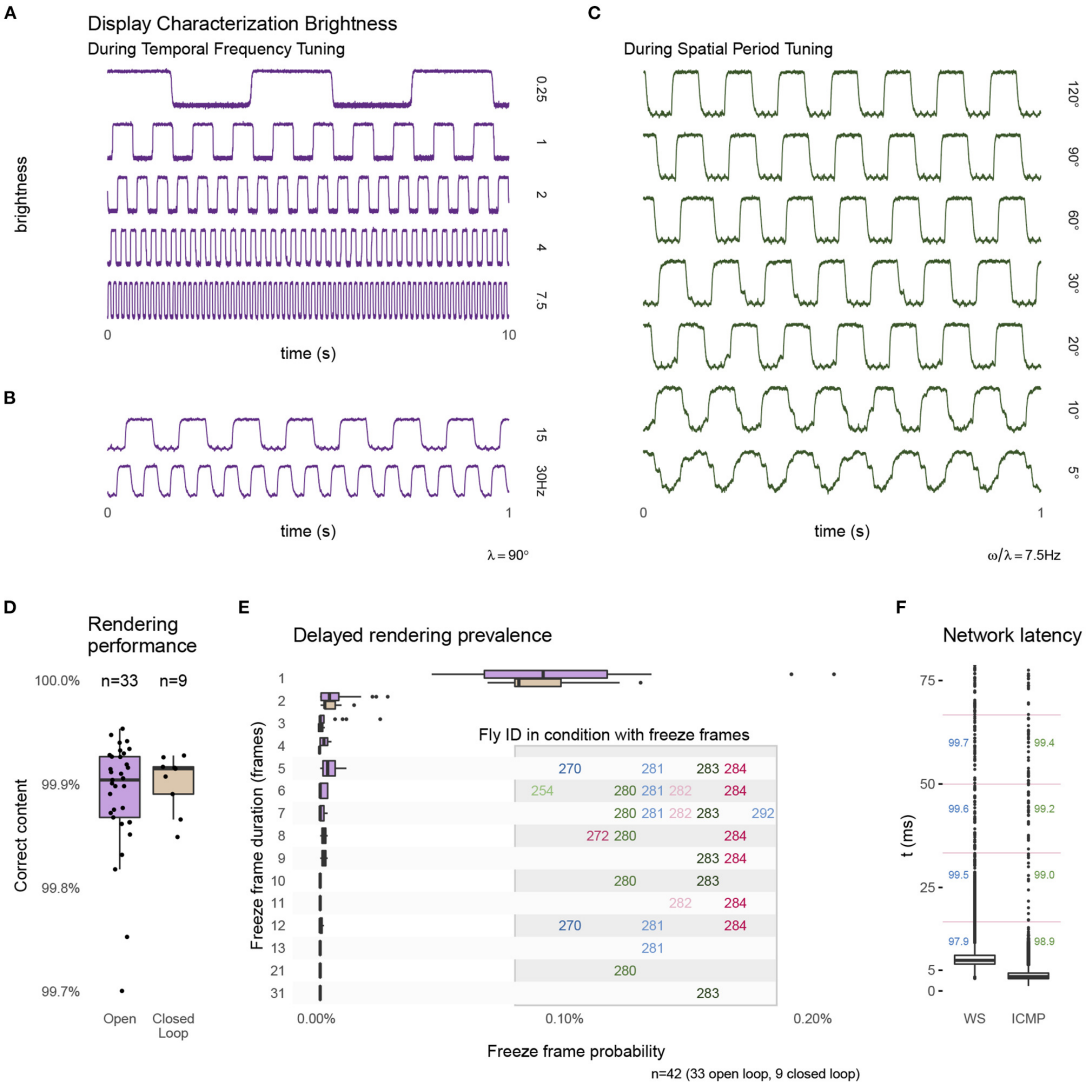
Technical performance of the experimental setup. During the presentation of moving grating patterns we measured changes in display brightness at the approximate location marked in Figure 3B. The measurements show a regular pattern that changes at the expected temporal frequency **(A,B)** and at the same temporal frequency of 7.5 Hz during conditions showing different spatial period grating motion **(C)**. The apparent filtering for the patterns with the finest bars is due to spatial averaging by the sensor. The traces show a small ripple of high frequency noise, this 60 Hz noise is in the sensor measurement and not due to the display. **(D)** The percentage of frames that were rendered correctly and within the expected time interval (see text for further details) during open-loop and closed-loop experiments. **(E)** Details of the frames that were not rendered within 1 frame interval shows a majority delayed by a single frame. A very small number of longer delays occurred, and all were from the same, few experiments. **(F)** The measured network latency for a round-trip message between FlyFlix server and client through WebSocket compared to a network ping (ICMP). The numbers mark the percentage of round-trips that would arrive within the 1st, 2nd, 3rd, or 4th ~17 ms display frame (indicated by the vertical lines). Box plots show the first and third quartile for the box, median for the center line, the whisker extend to 1.5 of the inter-quartile range (IQR). Panel **(D)** shows all data points, **(E,F)** only the outliers as individual points.

**Figure 5:**
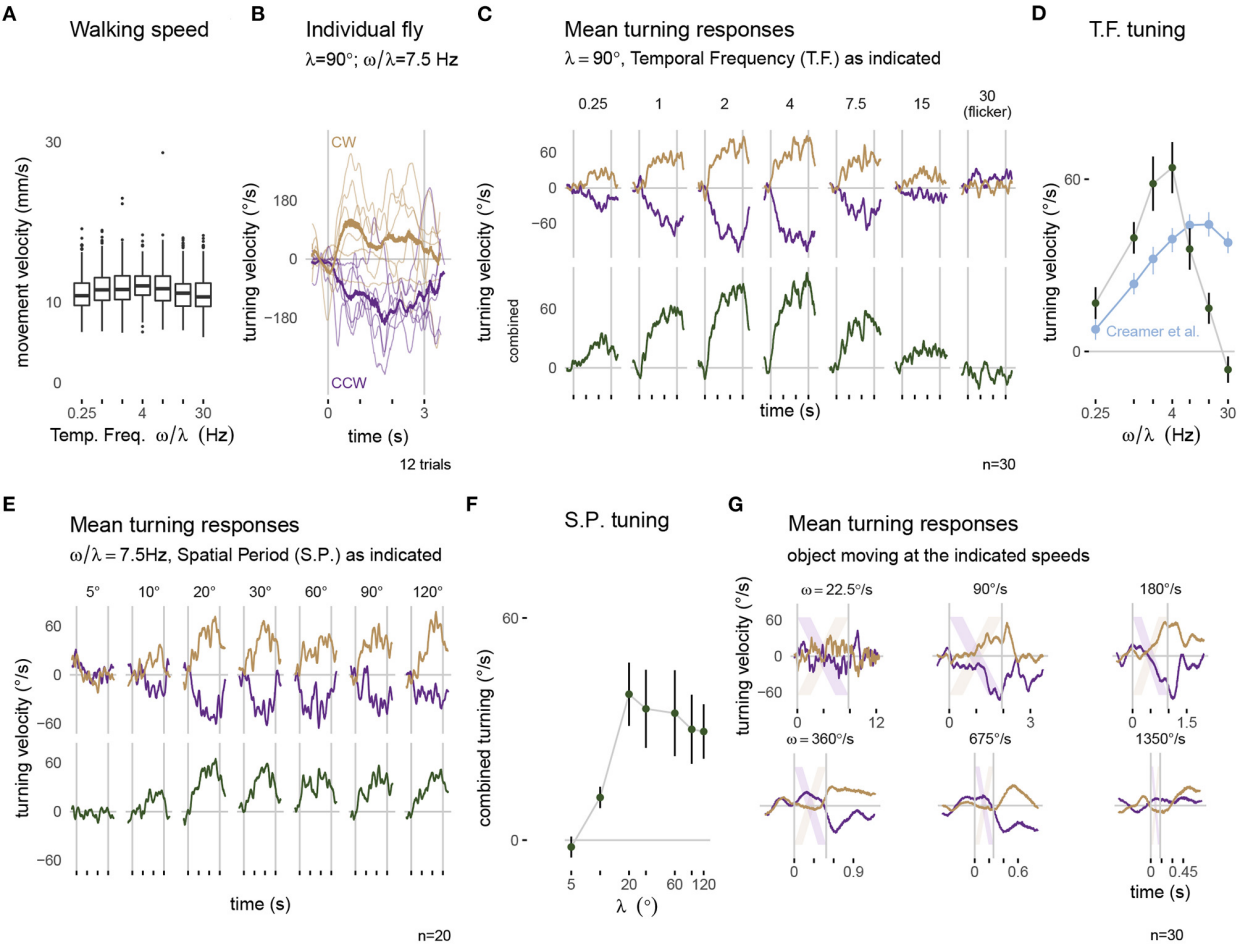
Visually guided turning behaviors measured with the optimized setup. Panels along the top row show walking behavior in response to a moving grating pattern with a spatial period of λ = 90°. **(A)** The forward walking speed of flies across the different conditions (*n* = 30). **(B)** The turning response of a single fly to multiple presentations of 3 s rotational stimuli moving clockwise (CW, yellow) and counterclockwise (CCW, purple). Thin lines represent single trial responses and the mean response across trials is in the thicker line. **(C)** The mean turning response across all 30 flies: the top row shows the responses to each direction of motion and the combined responses are plotted below. **(D)** The tuning curve summarizes the combined response, averaged during the period of stimulus presentation. For comparison, the data in blue is extracted from similar experiments on a different setup, from Figure 1H of Creamer et al. (2018). The mean turning responses for a series of gratings with different spatial periods are plotted in **(E)** and summarized as a tuning curve in **(F)**. **(G)** The mean turning responses to presentations of a single 45° bar sweeping across the display. The lightly colored stripes represent the expected position of the bar on the display. The box plot in **(A)** uses first and third quartile to span the box, 1.5·IQR for the whiskers and outliers are plotted as individual points. The error bar in **(D,F)** are plotted as mean ± SEM. A consistent color code for clockwise responses (yellow) and counterclockwise responses (purple) is used in panels **(B,C,E,G)**.

To our knowledge, inexpensive tablets have not been used to test detailed behavioral responses of flies to moving stimuli, and so we evaluated both the technical performance of the display system (Figure 4) as well as the behavioral responses of flies to tablet-displayed motion stimuli (Figure 5). Tablets featuring IPS displays with 60 Hz refresh rate are the most widely available inexpensive option. It will be interesting to reevaluate new display technologies (such as OLED) with higher refresh rates as these become less expensive. Our existing system could be rapidly adapted to using a student's personal smartphone instead of a tablet, further reducing cost (and probably distractions) in teaching environments.

#### 2.3.9. FlyFlix

By designing our inexpensive treadmill setup around a network-connected tablet as the visual display, we remove the need for any specialized devices for data acquisition or graphics cards for stimulus generation, but we developed software we call FlyFlix, to control experiments, generate stimuli, and log data. Figure 1C shows a simplified flow of information through the experimental setup. Our display connects through the web browser to the local URL of the FlyFlix server. Upon connection, the web server delivers the most recent version of the FlyFlix client software (written in JavaScript) as an HTML5 web page. The implementation follows an event-based approach with minimal dependencies between client and server, so that any device capable of displaying an HTML5 website can act as a client without prior installation of client software. We have verified that smartphones and computer monitors can be used to display the stimuli, but all results reported in this paper are from experiments using the tablet described in section 2.3.8.

The FlyFlix client and server communicate over a bidirectional, low-latency WebSocket connection. The server can deliver different experiments at specific URLs, or different views on the same experiment to different displays (a feature not used in our standard setup). Once the client connects to the server, it shows a “Fullscreen” and a “Start” button, the first changes the client to a full-screen mode, while the seconds sends the request to the server to start the experiment. After the protocol finishes or the WebSocket connection is interrupted, the FlyFlix client displays a button to “Reconnect” to the server. When the client starts the experiment, the server generates a set of trials based on the pre-specified configuration. The FlyFlix client renders a scene based on its local representation of the stimulus. The server sends updated parameters to change the representation and the client continuously reports back the actual state of the rendered stimulus. This bidirectional communication happens throughout the experiments with time-stamped messages. We implemented the FlyFlix server in Python-3.9 using the Flask-1.1.2 web framework. Bidirectional communication from the server-side is based on Flask-SocketIO-5.x with concurrent networking through Eventlet-0.30. The JavaScript client uses two external libraries: Socket.IO-3.1 for the communication and Three.js-r124 for rendering the visual stimuli.

For our “gold-standard” visually guided behavioral experiments, we wanted to present moving grating patterns composed of vertical bars across the display. Depending on the condition, we move distinct patterns at different speeds through the frontal visual field of the fly. Using the 3D graphics library Three.js, these stimuli are represented as segments of a virtual cylinder surrounding a virtual camera. We set the material of these segments to emits color but not be affected by virtual lighting. The virtual cylinder is 305 mm in diameter, matched to the size of a typical LED arena used in our lab, and a virtual height that exceeds the size of the virtual camera frame. The virtual camera accounts for the physical distance between the animal and display (35 mm), and has the effect of correcting the displayed size of the cylinder segments so that they span an equivalent azimuthal size from the fly's point of view, but span a different physical size on the display (illustrated in Figure 3B for a grating made up of 10° bars). We achieve the movement of gratings on the tablet by rotating the virtual camera although we can use FlyFlix to modify the virtual world as well, for example in more complex closed-loop conditions.

The FlyFlix server generates and controls experiments. Depending on the experimental condition, the server asynchronously sends parameters describing the virtual scene to the client. These parameters primarily concern the scene layout as well as rotational speed, orientation, and maximum refresh rate of the camera. Based on the set of parameters, the client continuously renders the current frame. This decouples the timing of server and client: the server communicates changes to the virtual world in real-time, but does not need to consider the capabilities of the client such as the screen refresh rate. Similarly, the client is independent from the server—if there is a lag in the communication from the server to the client, the client renders the previously communicated parameter instead of waiting for instructions. The client sends its time-stamped state to the FlyFlix server where they are stored in a log file together with the time-stamped server status. This on-line stimulus tracking allowed us to characterize the performance of our display and the network latency (Figure 4) and should enable powerful extensions of FlyFlix that we discuss below.

### 2.4. Experimental Protocol

To validate this new experimental setup we wanted to measure flies carrying out a well-studied visually guided behavior, the so-called syn-directional optomotor response, in which the flies steer, by turning, in the direction of a rotating visual pattern (Götz and Wenking, 1973; Seelig et al., 2010; Strother et al., 2017; Creamer et al., 2018). We recorded responses to open-loop stimulus presentations, in which the response of flies is measured, but not used to control the trajectory of the stimulus. To map the dependence of the turning response on the temporal frequency and spatial period of the pattern, we used periodic grating patterns moving at one of multiple (temporal) speeds, and a series of patterns composed of different grating (spatial) periods. In addition, we also measured object-following behavior, by recording turning responses to single sweeps of bright bars moving at different velocities.

For the temporal frequency tuning (14 conditions total), we showed grating with a spatial period of λ = 90° composed of pairs of alternating 45° bright and dark bars. The periodic pattern moves either clockwise or counterclockwise with one of seven angular velocities (ω = 22.5, 90, 180, 360, 675, 1350, and 2700°s^-1^). For a periodic pattern, the temporal frequency is the angular speed divided by the spatial period (ω/λ) and so the tested conditions include 0.25, 1, 2, 4, 7.5, 15, and 30 Hz. The 30 Hz stimulus serves as a control condition. For the spatial period tuning (14 conditions) we tested motion of gratings with one of 7 spatial periods (λ = 5, 10, 20, 30, 60, 90, and 120°) all at a temporal frequency of 7.5 Hz, spanning angular velocities between 37.5 and 900°s^-1^. At the beginning of each trial, the display shows the initial position of the pattern stationary for 500 ms, then moves for 3 s and shows the pattern stationary again for another 500 ms. Figure 3C shows some examples of the patterns displayed in these conditions.

In the object-following conditions, a bright vertical 45° bar moves across the screen, exactly once at one of 6 angular speeds (ω = 22.5, 90, 180, 360, 675, and 1350°s^-1^) in either the clockwise or counterclockwise direction. Consequently, these trials have different durations between 0.13 and 7.8 s. During the 500 ms pre- and post-trial period, the screen is fully dark. Diagrams in Figure 3D illustrate these conditions.

Open-loop conditions were interleaved with 3 s closed-loop trials, where the fly's turning controlled the position of the stimulus. We tested a variety of closed-loop conditions (data from these trials are somewhat ambiguous and not part of our analysis). For technical verification we extended closed-loop trials to a length of 30 s and also bracketed these trials between 500 ms pre- and post-trials. Within an experiment, each set of conditions was presented as randomly ordered blocks. The blocks repeated 6 times. The temporal frequency and spatial period mapping experiments were performed as separate protocols. At the beginning of each protocol there is a delay of 10 s to allow the experimenter to shield the experimental setup from the environment with a box, if desired. To minimize any unintended visual stimulation from the room, including any status lights on miscellaneous devices, we ran experiments in a darkened room, and a cardboard box, painted with BLK 3.0 on the inside, was placed over the experimental setup. All experiments were conducted on single tethered flies, with a target temperature of 32°C, to increase fly walking. Figure 5 presents the behavioral results.

### 2.5. Fly Preparation

We used the Dickinson Lab (DL) wild-type strain of *Drosophila melanogaster* for our behavioral experiments. This fly strain was established by interbreeding the progeny of 200 wild caught gravid females (Tammero and Dickinson, 2002). The original laboratory culture was maintained in Michael Dickinson's lab, from which the Reiser lab established a copy at Janelia in 2007. This strain has been used in dozens of behavioral studies and has been referred to as “DL” starting with Ofstad et al. (2011). For this study, we reared flies on standard cornmeal agar food at 21°C and 50 % humidity. We conducted the speed tuning (Figures 5A–D) and object tracking (Figure 5G) experiments on 30 female flies and the spatial period tuning experiments (Figure 5E) on 20 flies (randomly selected 11 male, 9 female). All flies were raised in a 16:8 h light-dark cycle; experiments were run on flies between 5 and 6 days post-eclosion.

Prior to tethering, we moved groups of ~4 flies from the fly vial to a 5 ml “Falcon”-style tube (12 mm wide) using a transfer funnel of our design (see Supplementary Figures S5A,B). The vial containing flies was placed in an ice bucket (not shown). After 5 min on ice, the immobile flies were carefully tapped onto the cold, temperature-controlled sorting platform (upper part of the red structure in Figures 2A,C). We used the suction from the fly picker (Figure 2E) to lift and then deposit each selected fly, one at a time, into one of the semi-cylindrical indentations in the lower part of the platform (see Figures 2A,C,D). It is occasionally possible to position a fly perfectly aligned into this “sarcophagus,” but typically flies need to be adjusted using a paintbrush. With a fine wire (see Supplementary Figures S5C,D) we placed a small drop of glue toward the anterior side of fly's notum, the dorsal surface of the thorax. The hand rest (see Figure 2A) supports the user's arm during these fine-scale manual steps. We used the three-axis linear stage Figures 2A,B to position the tether to just contact the glue. Once the “glue” is cured with short wavelength (UV) light, the micromanipulator was used to lift the fly out of the sarcophagus (Figure 2D).

We recommend a brief procedure of chilling flies. In our experiments, none of the tested flies were chilled for longer than 23 min. A reliable sign of vigorous flies and minimal effects of the chilling procedure is that flies should start moving within seconds of being removed from the sarcophagus—something we routinely observed. A body-fixed fly can be positioned in the experimental setup immediately, but we followed a standard practice of allowing for a 20–30 min recovery. Specifically, we placed the tethered flies upside down in a holding area for at least 30 min between tethering and the start of the experiment. We provided a piece of ~5 × 5 mm^2^ tissue to the flies, which they readily manipulated with their legs. This fly tethering procedure represents a practical compromise that works well in our experience—long enough to tether a small group of flies, while allowing them ample time to recover, but not so long as to compromise behavioral vigor.

### 2.6. Data Analysis

FlyFlix and FicTrac store the data in rectangular data files. While FicTrac follows a tidy data format, FlyFlix uses a key-value based long format. A custom Python script loads these different data formats into a consistent SQLite database. We use R-4.0 with the tidyverse-1.3 packages and ggplot-3.3 for data analysis and plotting.

Flies were presented with paired visual stimuli that moved in both the clockwise and counterclockwise direction. We recorded the ball rotation via FicTrac. Out of the 25 recorded variables, we used the “animal's heading direction (lab)” to estimate intended body yaw rotations and the “animal movement speed” for the walking velocity. We used “delta timestamp” to convert frame-based differences into time-based rotational velocity and the physical diameter of the sphere to calculate the movement velocity (Figures 5A,B). For time-series data, the average turning response was calculated for a sliding window of 5 camera frames across all trials of a condition. These responses were averaged on a per-fly basis (see Figure 5B), before being averaged across flies (top of Figures 5C,E,G). Responses to counterclockwise stimulus movement were scaled by −1 and averaged together with the clockwise responses for the combined responses (Figures 5C,E, bottom). The summary tuning curves (Figures 5D,F) show mean turning velocity during stimulus presentation as mean ± SEM across flies.

## 3. Results

### 3.1. Characterizing the Technical Performance of the Experimental Setup

As the inexpensive treadmill setup uses several components not typically used in animal behavior experiments, we measured many aspects of the system's performance, and summarize the results in Figure 4. To validate the tablet's display of our moving visual stimuli, we measured local brightness changes on one side of the display (position indicated on the right side of Figure 3B) with a mounted photo-diode (INL-3APD80, Inolux Corporation, Santa Clara, CA, USA). We viewed and logged the data on an oscilloscope (MDO3040, Tektronix Inc., Beaverton, OR, USA). Figures 4A,B shows typical measurements of the brightness changes measured for the moving patterns of the temporal frequency tuning conditions. Figure 4C shows typical measurements for moving patterns during the spatial period tuning conditions. These measurements suggest that the Fire 7 tablet reliably displays these periodic patterns, for example showing the expected periodic changes at the indicated temporal frequency. We note that even at the 30 Hz condition, which is half of the display refresh rate (lower trace in Figure 3B), the stimulus timing looks extremely reliable; this condition is included as a stimulus control, since at half of the display refresh rate, the display flickers, and thus produces no net motion. The spatial period conditions show a similarly reliable periodic pattern at 7.5 Hz (Figure 3C). The reduction in the sharpness of the edge transitions for smaller bars is simply due to spatial averaging by the sensor (and is a reasonable model for why the fly visual system also sees smaller period, thin-bar, patterns as consisting of lower contrast).

During experiments, the FlyFlix client records the rendering status for each frame. By providing this on-line stimulus tracking, not possible with many other display systems, a record of successful and delayed frames can be stored and incorporated into the *post-hoc* data analysis. Before the web-browser displays a frame, the software asynchronously requests an update of the rendered content based on the current set of parameters. If this request times out before the frame is rendered, then the previous content is shown again. Figure 4D shows the percentage of frames that are rendered correctly and within the allotted time, which is (on average) the inter-frame-interval of ~17 ms. We plot the percentage of correct frames for 33 open-loop experiments as well as from 9 closed loop experiments (for which the behavioral data are not shown). For both configurations, the average performance is quite reliable, with more than 99.9 % of the frames correctly rendered. Figure 4E provides details of the ~0.1 % of cases when frames were not rendered within this interval. We do not find any systematic errors. On average one out of every 1,000 frames skips exactly one frame update. Higher numbers of skipped frames are extremely rare, and tend to come in clusters, mostly during conditions with the same animals. Since FlyFlix records these measurements, trials above certain relevant thresholds can be identified *post-hoc* and removed from analysis.

Since the FlyFlix server and FlyFlix client communicate via a network, we characterized the latency of this asynchronous bidirectional communication by sending time-stamped packages from the server to the client, which immediately returns the package. In Figure 4F, we plot this WebSocket latency (WS) and also a ping using a lower network layer (ICMP). 97.9 % of the frames completed a round-trip within 1 inter-frame-interval (~17 ms, indicated with horizontal magenta lines in the plot) of the display, even though WebSocket based communication takes slightly longer with the additional protocol overhead. We expect that in our real application, the network reliability is even higher, since only half of a round-trip is required to update the display while the returned display state is not time critical.

In our experiments, we used our institute's infrastructure: the FlyFlix server was connected to a wired network, the tablet connected via Wi-Fi to a different subnet. Should the latency of an available network become too high, a local network router directly connecting FlyFlix server and client will improve the timing of the communication.

Taken together, the results of Figure 4, demonstrate that a low-cost tablet provides a reliable visual display producing excellent stimulus control and timing over measured system events. These technical measurements show that our low-cost system replaces many components typically required for precise experiment control (like data acquisition devices or high-end PC graphics cards) without sacrificing any performance, for the range of pattern speeds and network latencies described here.

### 3.2. Visually Guided Turning Behaviors Measured With the Optimized Setup

An important demonstration of our new, integrated system is that “typical” fly behaviors can be measured from flies tethered using our new tethering station and behavioral data collected using the new experimental setup. We focused on the optomotor responses, and present results from (30+20 =) 50 flies across 2 different protocols (detailed in sections 2.4 and 2.5). Figure 5A shows the walking speed of flies during each trial of the temporal frequency protocol. Across conditions, flies walk with a similar speed, with a mean around 10 mm s^-1^ which is slightly faster than walking speeds measured in other fly-on-ball experiments (Creamer et al., 2018), and is only slightly slower than the walking speeds of freely walking flies at similar temperature (Ofstad et al., 2011).

When presented with rotating patterns, flies tend to turn in the direction of the pattern movement, a response seen in single trials and across trials for the example condition shown in Figure 5B. While there is some trial-to-trial variability, in nearly every trial, the flies turned in the clockwise, or positive direction (in yellow) for clockwise pattern motion and in the counterclockwise, or negative direction (in purple) for counterclockwise pattern motion, a pattern that is clearly seen across flies and stimulus speeds (top of Figure 5C). The amplitude of the turning velocity we measured depends on the temporal frequency of the pattern movement (observable in the data combined from both directions, in the lower row of Figure 5C). This is precisely the expected result, since temporal frequency tuning is a well-described aspect of fly motion vision—insects are most sensitive to movement of periodic pattern with some temporal frequency optimum, and are less sensitive to movements with both higher and lower temporal frequency (Götz and Wenking, 1973). We compare our results, plotted using the mean responses during the period of stimulus presentation as a tuning curve, to the most relevant, recent independent measurement from another lab using a different setup (Figure 5D contains an overlay of data from Creamer et al., 2018). We find that in our experiments, for most conditions, flies turned more overall, and we see similar, monotonically increasing response levels up to 4 Hz motion. At the highest temporal frequencies we see an interesting difference, where our responses were reduced, the responses from Creamer et al. (2018) remain much larger. We attribute this difference to limitations of our display. As previously discussed, the tablet refreshes the screen content with 60 fps; at this refresh rate, a 30 Hz temporal frequency motion grating will appear as flicker—containing no net motion, and so it is expected that our flies cannot turn to follow motion that is not there. Similarly, the responses to 7.5 and 15 Hz pattern motion are reduced since the illusion of smooth motion is weaker at these speeds. Aside from these technical limitations of the display at very fast speeds, we find excellent concordance between our measurements and those of previous experimenters.

The optomotor turning response is also expected to depend on the spatial period of the grating pattern (Buchner, 1976; Creamer et al., 2018). We presented a series of grating patterns with different spatial periods at a fixed temporal frequency of 7.5 Hz. The flies responded with large, consistent turning to patterns with a grating period above λ = 20° (Figures 5E,F). For narrower stripes, the responses were reduced, and in fact no consistent turning was measured for the pattern with λ = 5°. This result is expected based on prior work, and is remarkably similar to the measurement of Buchner (1976), who used a very different stimulus strategy.

Finally, we tested the flies' ability to track a moving bar, a behavior that is known to depend on both the motion and position of the moving object (Poggio and Reichardt, 1973; Bahl et al., 2013). As with the rotating grating patterns, we found that flies turned so as to follow the direction of the rotating bar (Figure 5G). The peak turning velocity was similar between different rotational velocities of the stimulus, and quite similar to peak turning during the grating motion. To casually explore the position-dependence of the turning response, it suffices to note that most of the turning reaction occurs once the object (position indicated by the diagonal lines) crossed the midline (most notable for ω = 90, 180, and ~360°s^-1^). It is as if the flies don't attempt to orient toward an object they are likely to intercept as it approaches their midline, but once an object is getting away (as measured by its progressive, or front-to-back motion), their attempted tracking behavior rapidly increases. This response profile matches the recent measurements of walking flies (Bahl et al., 2013), but differs somewhat from the behavioral reactions of tethered flying flies that respond to both the regressive and progressive motion of the object (Reiser and Dickinson, 2010). For the fastest speeds tested, the flies were unable to track, that is “catch up to” the spinning bar, and the responses are seen to lag the position of the stripe by more than 100 ms. In the condition with ω = 1,350°s^-1^, the object moved across the 60 fps display in less than 8 frames with displacements of over 20° between frames, which are too large for the fly to smoothly integrate as motion, and as expected the flies barely turned to this condition.

In Figure 5, we summarize the behavior of *Drosophila* in our optimized, inexpensive treadmill setup, in a sophisticated range of stimulus conditions. We show clear symmetric turning responses to all symmetric stimulus conditions. The temporal frequency and spatial period tuning as well as the object tracking behaviors are highly similar to previously published measurements from other labs using different experimental setups, for all but the fastest stimulus conditions. Based on these results, we unreservedly recommend this low-cost setup, not only for teaching purposes, but for nearly any research application.

## 4. Discussion

In this paper, we have described our re-implementation of a complete system for tethering flies and the accompanying experimental setup for measuring tethered fly walking behavior to controlled visual stimuli (Figures 1, 2). Our spherical treadmill setup takes a fresh look at the fly-on-a-ball paradigm. While the design is guided by several decades of experimental methods development, we have been optimizing the setup by simplifying the components, reducing costs, and ensuring availability. Since many of the components have not previously been deployed in animal behavior setups, we validated their performance (Figure 4). We found excellent reliability for the low-cost display and low network latencies, which combine to establish a highly reliable new method for experimental control. This system comes with other advantages such as a flexible stimulus control software that can dynamically correct for the viewing angle (Figure 3). Finally, we measured the walking behavior of flies to a range of moving visual stimuli and confirmed, in exquisite detail, that our new setup is capable of reproducing nearly all relevant prior measurements using similar visual stimuli for wide-field gratings and small moving objects. Therefore we now have a low-cost setup that is a quite reliable instrument, and consequently find that it produces highly reliable open-loop behavioral measurements. Based on this experience, we believe our setup will be ideal for teaching courses and for a wide range of laboratory uses. We sincerely hope that the reduced complexity and enhanced accessibility of these setups will excite many young scientists about quantitative animal behavior, and will increase the reproducibility of research observations. In the following sections we discuss cost savings of our system, the cost of cost savings in the form of limitations, some possible extensions, and future work.

### 4.1. Costs and Availability

We have endeavored to reduce the cost of the system at each step, often with considerable cost savings relative to alternative contemporary setups. We estimated the costs based on building a single setup, using parts available in the U.S., during the spring of 2021. Many of the components are available as generic parts from multiple vendors, and most will also have comparable alternative, if not identical, components available world-wide. We selected example sources to illustrate the price range for potential cost savings and overall costs and provide website links for the same purpose. We give examples and not endorsements for or against particular vendors. We estimate the prices for 3D-printed components using the online instant quote at https://craftcloud3d.com. For the laser cutting, we use estimates from https://ponoko.com. We base our cost estimation of consumables and commodities like glue, tethers, and screws on a projected weekly consumption. In Tables 1, 2, we link to packages that will last for longer periods of time. Those with access to a 3D printer, a laser cutter, or a selection of screws can expect overall lower costs.

**Table 1:** Price estimation of parts for experimental setup.

**Part**	**Description**	**Link**	**Price**
Sphere	Milled or filed	generalplastics.com	
Display	Amazon Fire Tablet	amazon.com	$50.00
Baseplate	Acrylic Material only	mcmaster.com	$13.00
Rubber feet	for Baseplate	amazon.com	$ 11.00
Heat-pad	70mm	amazon.com	$ 15.00
Temperature control	Heat-pad thermostat	amazon.com	$5.00
Camera	PS3 Eye	ebay.us	$15.00
Micromanipulator	3D-printed (ABS)	reiserlab.github.io	$20.00
Screw	M3x0.5 40mm	mcmaster.com	$0.49
Locking nuts	M3x0.5	mcmaster.com	$0.11
Nuts	M3x0.5	mcmaster.com	$0.06
Washer	M3	mcmaster.com	$0.23
Sphere holder	3D-printed (ABS)	reiserlab.github.io	$6.00
Sphere holder post	3D-printed (ABS)	reiserlab.github.io	$5.00
Lamp post and shade	3D printed (ABS)	reiserlab.github.io	$15.00
IR LED	940 nm 5 mm LED	digikey.com	$0.90
Power supply	Any 5V power source	adafruit.com	$8.00
Tube Clamp	Keck Roller Clamp	usplastic.com	$5.00
Lens	25mm M12 Lens	m12lenses.com	$24.25
Lens holder	M12 Lens holder	m12lenses.com	$3.00
Lens extension	M12 Macro Extension	m12lenses.com	$3.00
Tablet holder	any	amazon.com	$ 17.00

*Usually one item per line is required, but commodities like nuts are available in packages that will supply components for several setups. For package prices and more details refer to the text and Supplementary Tables S1–S4*.

**Table 2:** Suggestion for components in an inexpensive treadmill tethering station.

**Part**	**Description**	**Link**	**Price**
microscope	any dissecting scope		
glue	glass to glass adhesive	kemxert.com	$0.58
UV protective glasses	Different manufacturers	amazon.com	$11.00
Fly picker wand	Transfer Pipette	amazon.com	$0.03
Temperature control	Chiller thermostat	amazon.com	$5.00
Tether	Dispensing needle 34GA	bstean.com	$0.96
Funnel	3D-printed (PLA, ABS)	reiserlab.github.io	$3.00
Micromanipulator	3D-printed (ABS)	github.com	$20.00
Screw	M3x0.5 40mm	mcmaster.com	$0.49
Locking nuts	M3x0.5	mcmaster.com	$0.11
Nuts	M3x0.5	mcmaster.com	$0.06
Washer	M3	mcmaster.com	$0.23
Heat pump	Peltier on heat sink	adafruit.com	$35.00
Power Supply	12V 5A (Heat Pump)	adafruit.com	$25.00
Sarcophagus	3D-printed (ABS)	reiserlab.github.io	$3.00
Thermal Tape	for Sarcophagus	adafruit.com	$ 0.95
Tether Station holder	Laser cut (acrylic)	reiserlab.github.io	$15.00
UV Curing light	UV Keychain light	amazon.com	$1.33
Paintbrush	Fine tip	amazon.com	$7.00
Round bottom tube	Chilling tube	mcmaster.com	$0.06
Heat sink holder	Hand rest	ponoko.com	$18.00
Hollow Body Pin Vise	Flyhook holder	mcmaster.com	$16.00

*Usually one item per line is required, but commodities like nuts are available in packages that will supply components for several setups. For package prices and more details refer to the text and Supplementary Tables S1–S4*.

**Figure 6:**
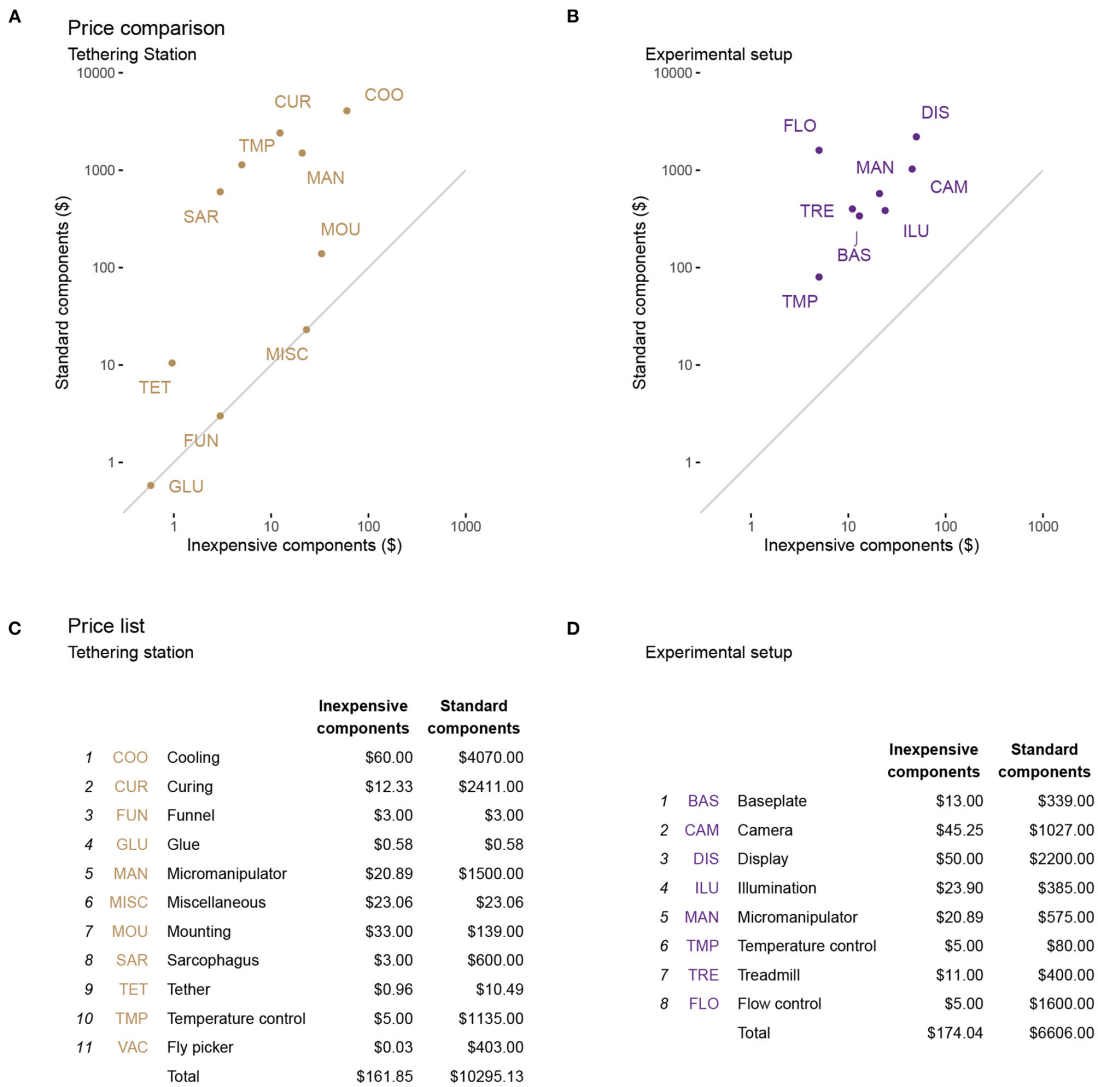
Estimated cost savings for each setup. The price of each functional unit is a comparison between the standard setups and the optimized, inexpensive tethering station **(A)** and experimental (treadmill) setup **(B)**. The diagonal line in each of (a), (b) represents an equal price in both setups. In **(C,D)**, we list the components (or functional units) represented by the labels in **(A,B)**. We use a projected maximum of weekly consumptions for expendables (e.g., glue and tethers). Between the two versions of these systems, we estimate a ~50-fold cost saving. Further details are provided in the text and in Tables 1, 2, detailing these components.

For the comparison to a contemporary setup, we surveyed several groups and specified a system that would realistically represent the type of setup we would build in our lab today for ongoing research projects. Below we detail a few key components, and summarize the systems' cost in Tables 1, 2, and in Figure 6). Figure 6 shows we can assemble both complete systems for ~$330, whereas the standard, yet very nice, pair of setups would cost ~$17,000, a remarkable ~50-fold cost reduction.

One simple way to reduce costs and increase access is to exclusively use Free Software and other open-source components. From GNU/Linux as the operating system, to FicTrac, camera drivers, FlyFlix, and Firefox, all are available without paying software license fees. Furthermore, the majority of the components in the Component-Designs GitHub repository are constructed using Free Software such as FreeCAD, KiCAD, and Inkscape. As a direct consequence, the software necessary to modify our designs is available without hidden costs and for all major operating systems in the foreseeable future. Communities around these software packages provide good documentation, tutorials, and support for any type of questions. In the long term, open standard file formats used by Free Software also ensure unrestricted exchange of design files, beyond the specific software packages we used.

### 4.2. Trade-Offs and Limitations

The flexibility and modularity of our proposed system is also a limitation: it takes more time and effort to make and assemble the systems based on components from multiple vendors, rather than ordering ready-made products. We sought to replace all custom parts with commercially available inexpensive components wherever possible, such as the display system or the tethers, but in many cases, no alternative existed and we turned to custom designs.

Many components of our setup are produced in a 3D printer or a laser cutter. This may increase access compared to custom-machined metal parts, but it is still a limitation. Nevertheless, we see three main alternatives to produce these parts: (1) high quality 3D printers are becoming more affordable and easier to use, (2) maker spaces provide access to 3D printers in communities across the world, and (3) many companies offer 3D printing as a service. We used the third (and most expensive) option in our cost estimates (Figure 6). We consider access to a laser cutter as nice, but unnecessary for building this setup (alternatives discussed throughout). The factors regarding price, maker spaces, and online services also apply to laser-cutting acrylics. Building a new experimental setup is always a time-consuming endeavor, but even more so when the components need to be built from scratch. We estimate ~5 h of printing time on the Stratasys F-170 printer, but could take considerably longer on the more common, less expensive printers. Potentially the use of 3D printing services is an option to reduce print time and the initial expertise and equipment required. We further estimate that another ~5 h are necessary for assembling the first setup. In the near future, we will provide printing and assembly advice on our accompanying repository based on feedback from early adopters.

FlyFlix, the system of a single server providing stimuli for network connected display clients, is extensible to multiple tablet displays. For the low-cost implementation described here, we have only used a single display in front of the fly covering ~130° in azimuth and 100° in elevation. Our lab's standard cylindrical displays cover 270° in azimuth, and this larger field of view is critical for some visually guided behaviors. Virtually any display will present non-uniform brightness from the perspective of the fly. In our current implementation, we do not correct for this, as there is little evidence to suggest that optomotor behaviors with large-field, high-contrast gratings are sensitive to these local brightness variations. Nevertheless, the brightness of the display as viewed by the fly, at each location on the screen, can be measured and corrected for by non-uniformly masking the local brightness of the display. This step should be seriously considered if users wish to use such a display for measurements of neuronal responses within small receptive fields. Furthermore, the tablet we chose only supports refresh rates of 60 fps. This limits the speed of stimuli that can be shown, including to motion speeds that the fly can perceive (see Figure 5D). Many apparent motion stimuli—including most of the moving gratings and the small moving objects shown in Figure 5—can be very well-approximated at this display refresh rate, but this illusion of smooth motion breaks down for stimuli defined by very fast motion. Newer handheld displays with higher refresh rates and gaming monitors used in other experimental setups overcome this limitation, but at significantly increased cost (Kaushik et al., 2020). The FlyFlix software is agnostic to the display and should work “out of the box” with higher refresh rate displays. Nevertheless, network latency will be a limiting factor for high-speed closed-loop systems, but there is little reason to believe that flies (or just about any animal) required closed loop latencies that are less than ~10 ms.

### 4.3. Extensions and Future Work

The challenge of setting up multiple rigs in a teaching lab to provide hands-on experience with *Drosophila*'s fascinating walking responses to visual stimuli initially inspired the inexpensive treadmill project. Since then, we optimized the setup and so far only tested it with fruit flies. Nevertheless, we expect that adapting the setup to other insects should be straightforward. The Sarcophagus already accommodates many body sizes and could be modified for others. A much larger insect may require a larger ball size, but fortunately, the nature of our manufacturing process and the availability of our 3D designs allows any components to be scaled to adapt to specific animal sizes.

While we have focused on visually guided behaviors with this setup, it would be very exciting to implement other types of sensory stimulation: wind, humidified air, sounds, odors, or even polarized light (Mathejczyk and Wernet, 2020). The inexpensive treadmill setup could readily be applied to longer duration observational studies of individual flies, for example in sleep studies or starvation experiments. All of these can be integrated into our experimental design with little to no modification to the existing components.

While we have achieved all of our initial goals, we continue working to improve the system. In the near future, we plan to provide more accessible alternatives to our hand-filed balls and a suitable replacement for laboratory wall air to float the ball. On the software side, we will continue to expand the capabilities of FlyFlix. One exciting direction is to use the on-line stimulus tracking to allow instant verification, and for example, to automatically repeat any trials during which stimuli were not successfully presented. Another important improvement for the combination of FlyFlix and specific tablets will be incorporating stimulus calibration information. One important goal will be to compensate for the brightness of the display, at different locations, and possibly for different color channels, to achieve a more uniform luminance distribution from the fly's perspective.

The open-loop experiments detailed in Figure 5 show that our new system is capable of replicating a wide range of visually guided behaviors in walking flies. In addition, we have implemented closed-loop protocols and confirmed that they are technically working. So far we have not been impressed with the behavioral results from this subset of closed-loop trials and so we continue to optimize these experiments and hope to report robust closed loop behaviors in the near future. Finally, we will implement a low-cost solution for optogenetic stimulation of walking flies, and will adapt the setup as needed so that we can mount it under a microscope to accommodate electrophysiology or calcium imaging measurements. We will post all updates on the accompanying repository and we welcome all feedback, ideas, and contributions.

## Data Availability Statement

All files and code for designing and constructing an Inexpensive Treadmill setup is available as part of the online repository https://reiserlab.github.io/Component-Designs/. Upon request, the authors will provide data from the technical and behavioral verification.

## Author Contributions

MR and FL conceived of the project and wrote the paper together. FL developed methodology, wrote software, carried out experiments, and analyzed the data. Both authors contributed to the article and approved the submitted version.

## Conflict of Interest

The authors declare that the research was conducted in the absence of any commercial or financial relationships that could be construed as a potential conflict of interest.
