# Supplementary material for: An Inexpensive, High-Precision, Modular Spherical Treadmill Setup Optimized for Drosophila Experiments
Source: Front Behav Neurosci. 2021 Jul 16;15:689573. doi: 10.3389/fnbeh.2021.689573 (PMC8322621; doi:10.3389/fnbeh.2021.689573)
Supplement: Supplementary file 1 [file Data_Sheet_1.pdf]

Supplementary Material for ‘An Inexpensive Treadmill Setup for *Drosophila*’

1 FIGURES

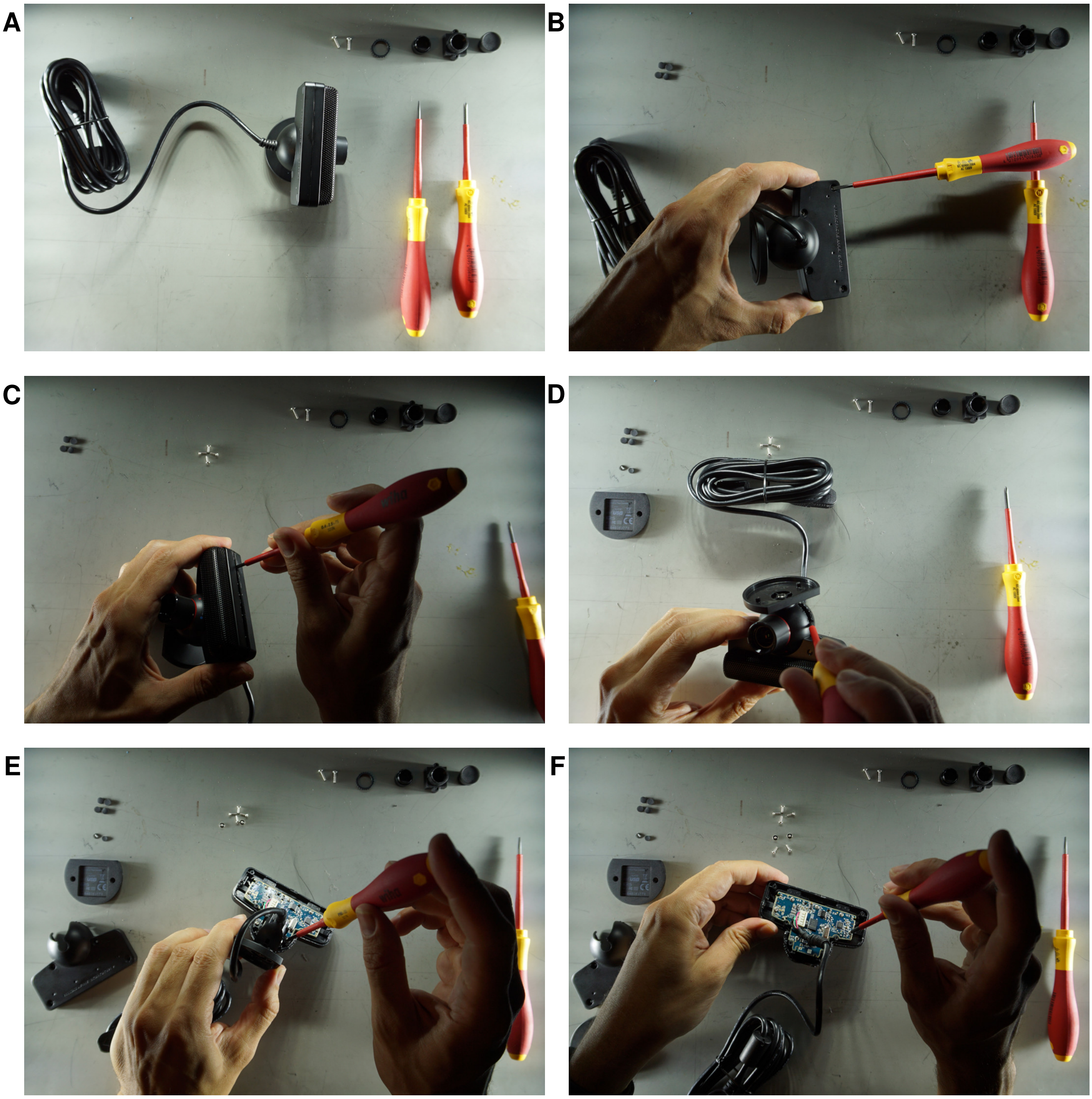

Figure S1. Disassembling the camera (A) Tools and parts required for modification. **(B)** Unscrew the back of the camera. **(C,D)** Carefully break notches holding back and front together. **(E,F)** Unscrew cable holder and PCB.

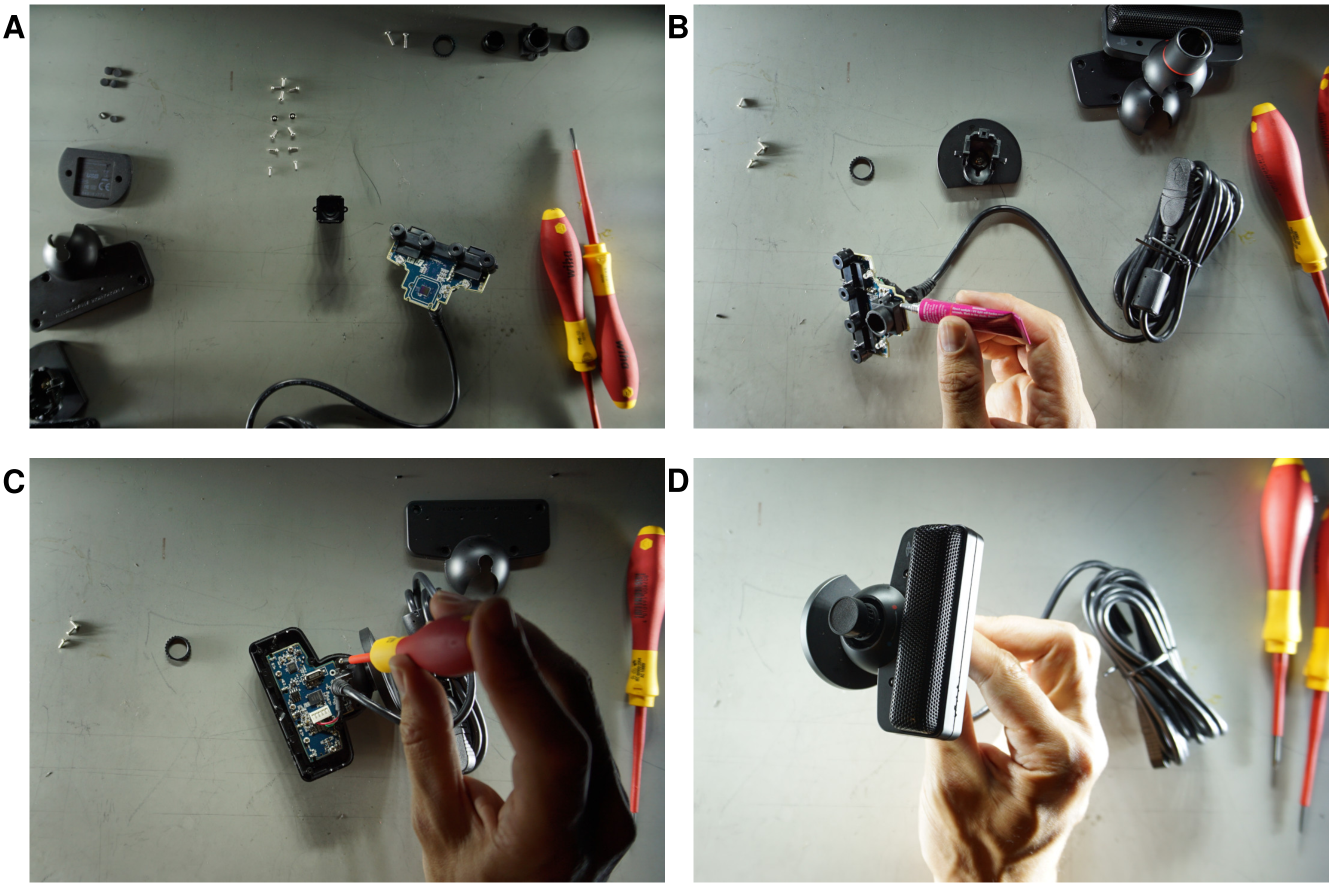

Figure S2. Modifying the camera (A) remove the lens from the front of the PCB. **(B)** Attach the M12 lens mount in the same place. **(C)** reattach PCB to camera case. **(D)** Close camera case. Without the broken notches removed in Figure S1C, some glue is needed. In the picture, the macro lens has a lens cover.

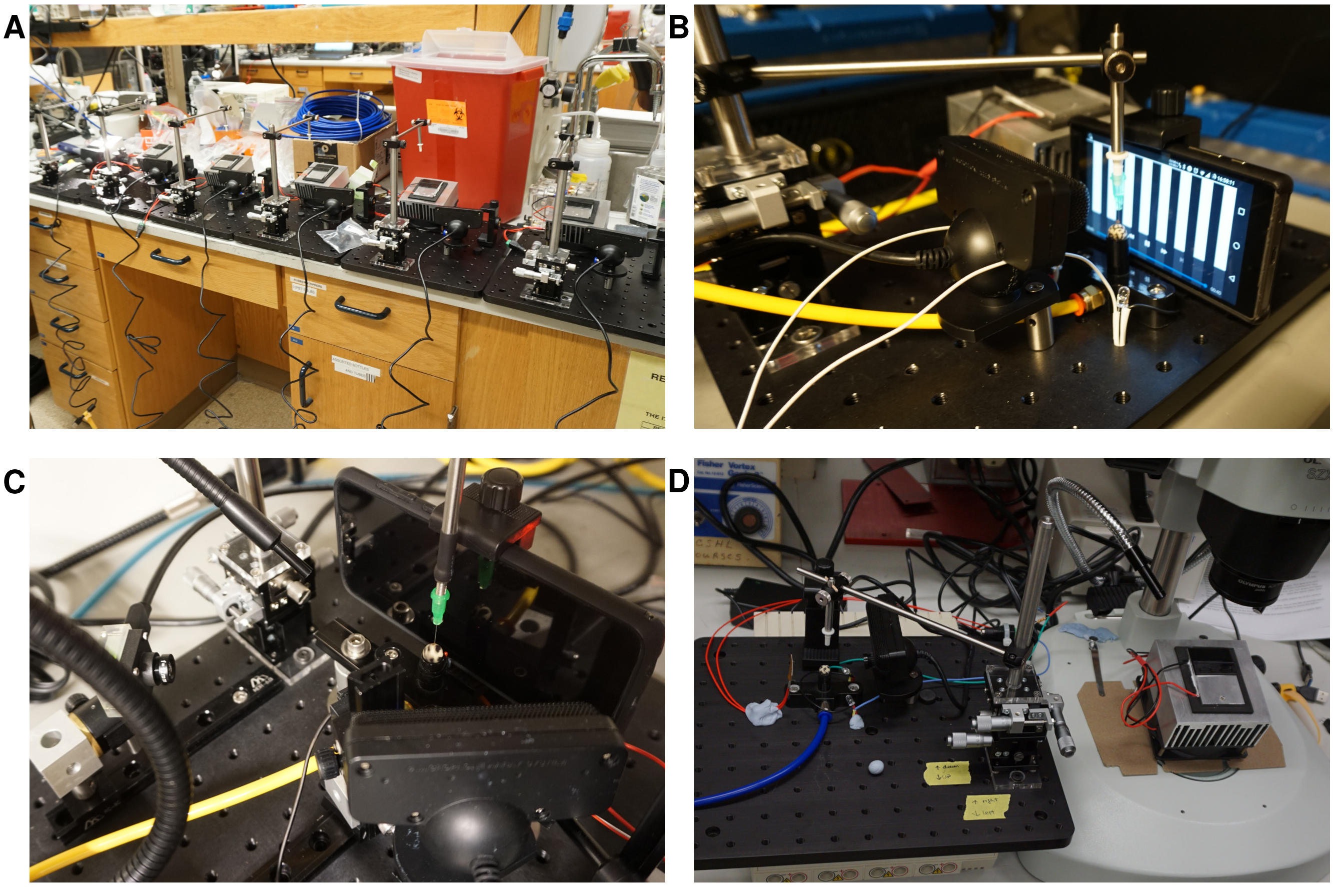

Figure S3. Early versions of these setups (A) 6 setups used in the CSHL course. **(B)** Shared Micromanipulator in combined tethering and experimental setup. **(C)** Prototyping camera angles and display sizes. **(D)** Shared Micromanipulator between tethering and experiment, for a heating setup, without the display.

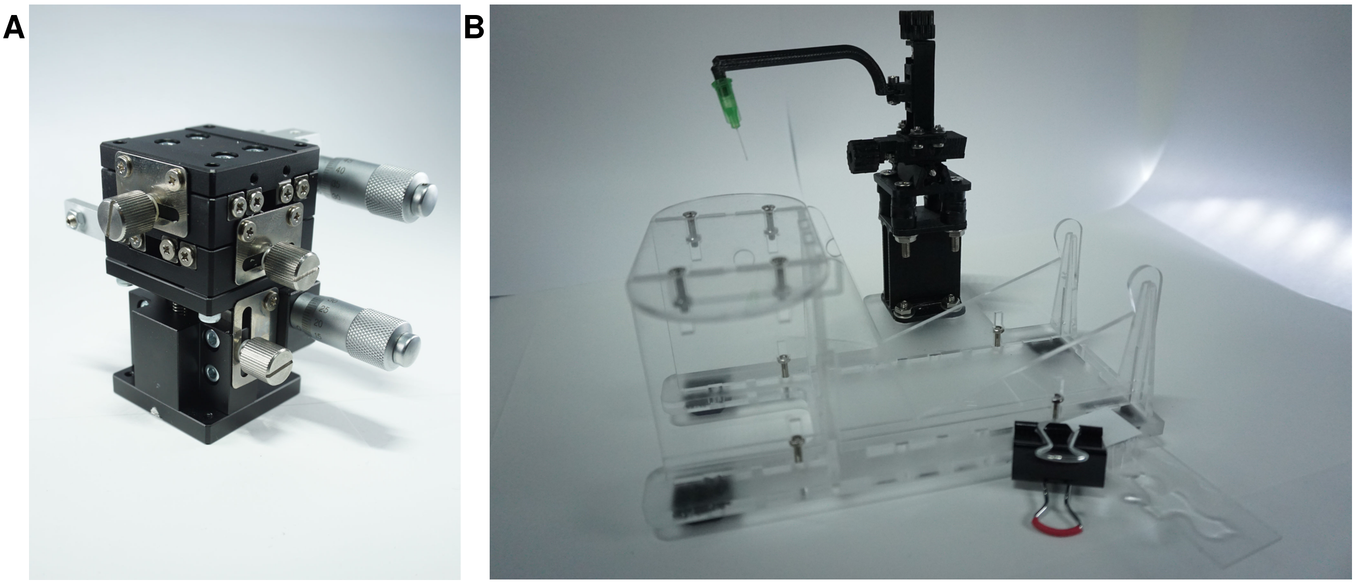

Figure S4. Micromanipulators (A) LD40-LM micromanipulator from overseas distributor. **(B)** Arm rest with fixture to hold the Chiller at the ideal angle, cut from acrylic. A glass slide for holding a few drops of glue is in the foreground and the 3D-printed micromanipulator in the background.

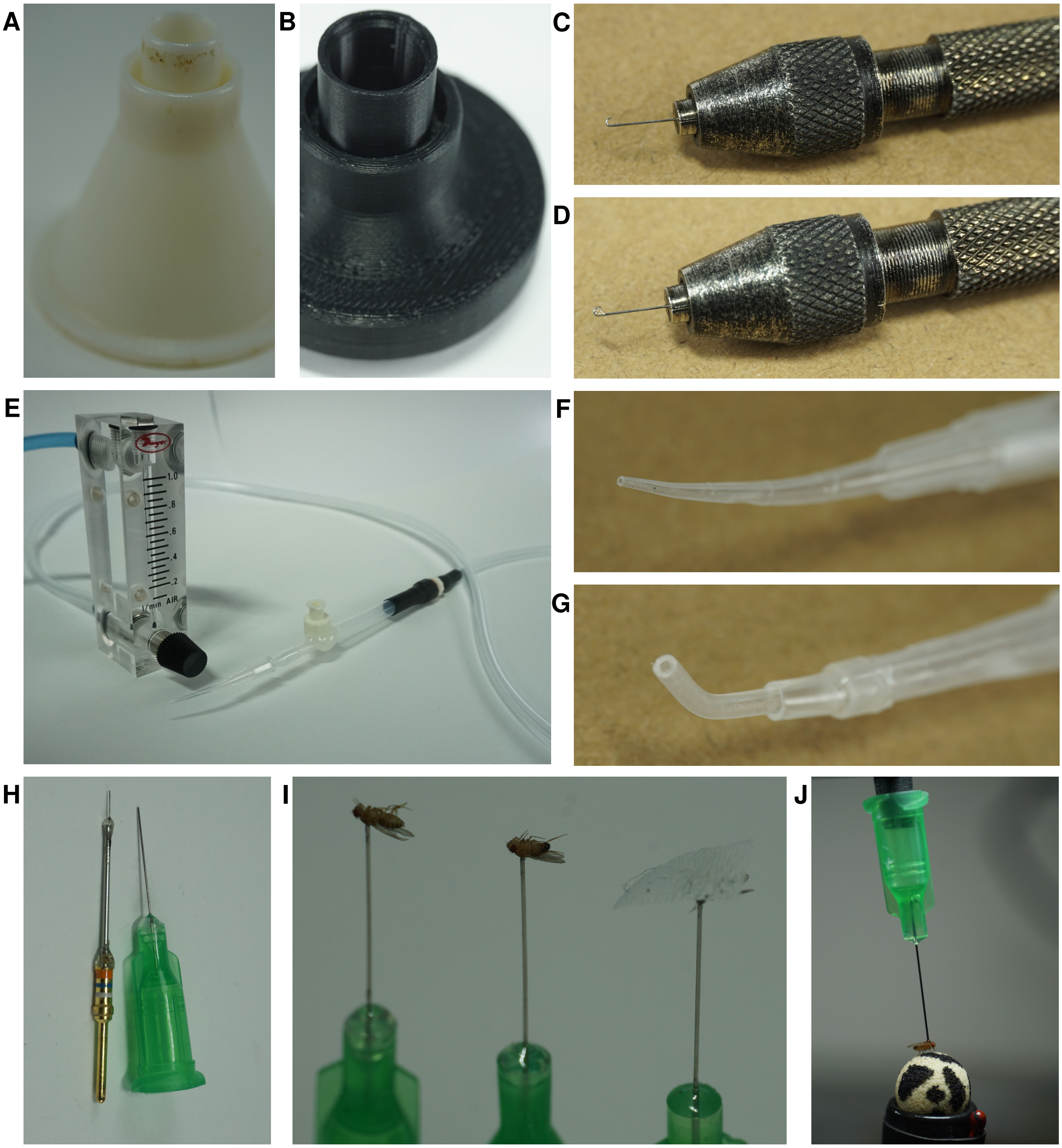

Figure S5. Gallery of useful tools **(A,B)** 3D-printed funnels used to move flies between bottles and vials. A bent minutien pin used to move the fly in the Sarcophagus and apply glue, without glue **(C)** and **(D)** with glue. **(E)** Flowmeter to regulate negative pressure for the fly picker. Tip of the fly picker made from micropipette tip **(F)** and shrink tube **(G)**. **(H)** Traditionally made tether (left) and blunt dispensing tip with Luer lock alternative (right). **(I)** tethered flies in holding area prior to experiments, the 3rd holding a piece of tissue. **(J)** Body-fixed, tethered fly on a ball, showing the modest 'uphill' orientation of the fly.

2 TABLES

Table S1: Components used in the tethering station for the inexpensive treadmill. The exact links and prices may vary, but with the product number (PN) and description, other vendors and similar products can be found.

part	description	link	PN	price
microscope	various; any dissecting scope			
glue	glass to glass adhesive	http://www.kemxert.com/product-catalog.cfm?pg=product-catalog&prd_pct_id=2	KOA-300	$30.00
UV protective glasses	different manufacturers	https://amazon.com/dp/B01KJ9JKB6		$11.00
Fly picker wand	Transfer Pipette	https://amazon.com/dp/B07RFFMKBM		$10.00
Luer connector	Fly picker component	https://www.mcmaster.com/51525K425/	51525K425	$5.00
Pipette tip	Fly picker tip	https://www.mcmaster.com/7015T41/	7015T41	$5.20
Temperature control	Chiller peltier thermostat	https://amazon.com/dp/B07VDRGK9F	XH-W1209	$5.00
Tether	Blunt tip dispensing needle 34G	https://www.bstean.com/product/industrial-unsterilized-blunt-tip-dispensing-needle-with-luer-lock-34-ga-x-1-2-50-pcs/	AG-ABSS-99D0	$16.00
Funnel	3D printed (PLA, ABS)	https://reiserlab.github.io/Component-Designs/tether/funnels		$3.00

Continued on next page

Table S1: *(continued)*

part	description	link	PN	price
Micromanipulator	3D printed (ABS)	https://reiserlab.github.io/Component-Designs/tether/micromanipulator		$20.00
Screw	M3x0.5 40mm	https://www.mcmaster.com/91287A028/	91287A028	$8.09
Locking nuts	M3x0.5	https://www.mcmaster.com/90576A102/	90576A102	$3.57
Nuts	M3x0.5	https://www.mcmaster.com/90592A085/	90592A085	$0.88
Washer	M3	https://www.mcmaster.com/95610A530/	95610A530	$3.81
Heat pump	Peltier element on heat sink	https://www.adafruit.com/product/1335	1335	$35.00
Heat pump power supply	12V 5A	https://www.adafruit.com/product/352	352	$25.00
Thermal Tape	to fix sarcophagus on peltier	https://www.adafruit.com/product/1467	1467	$0.95
Sarcophagus	3D printed (ABS)	https://reiserlab.github.io/Component-Designs/tether/sarcophagus		$3.00
Tether Station holder	Laser cut (acrylic)	https://reiserlab.github.io/Component-Designs/tether/station		$15.00
UV Curing light	UV Keychain light	https://amazon.com/dp/B086PW9DBH		$8.00
Paintbrush	fine tip	https://amazon.com/dp/B0878MN2VR		$7.00

Continued on next page

Table S1: *(continued)*

part	description	link	PN	price
Round bottom tube	Chilling tube	https://www.mcmaster.com/7012A54/	7012A54	$15.00
Heat sink holder	Hand rest	https://www.ponoko.com/materials/anti-static-acrylic		$18.00
Hollow Body Pin Vise	Flyhook holder	https://www.mcmaster.com/8455A18/	8455A18	$16.00
Minutien Pin	Flyhook	https://amazon.com/dp/B00J5PMPJA		$17.00

Table S2: Components used in the inexpensive treadmill experimental setup. The exact links and prices may vary, but with the product number (PN) and description, other vendors and similar products can be found. Some parts are listed as alternatives, for example the commercially available LD40-LM micromanipulator and the 3D-printed one.

part	description	link	PN	price
Sphere	milled or filed	https://www.generalplastics.com/products/fr-7100	FR-7120	
Display	Amazon Fire Tablet	https://amazon.com/dp/B07FKR6KXF	KFMUWI	$50.00
Tablet Holder	any holder suitable for 7" tablet	https://www.amazon.com/dp/B08F77HBSB	B08F77HBSB	$13.00
Baseplate	Acrylic Material only	https://www.mcmaster.com/4615T37	4615T37	$13.00
Baseplate	5.5mm acrylic with holes	https://www.ponoko.com/materials/black-acrylic		$27.00
Micromanipulator	XYZ micromanipulator	https://www.aliexpress.com/item/33013923564.html	LD40-LM	$80.00

Continued on next page

Table S2: *(continued)*

part	description	link	PN	price
Flow meter	Dwyer Flowmeter	https://www.dwyer-inst.com/Product/Flow/Flowmeters/VariableArea/SeriesVF	VFA-23	$40.00
Heat-pad	70mm round	https://www.amazon.com/dp/B07P1H8N8H		$15.00
Temperature control	Heat-pad thermostat	https://amazon.com/dp/B07VDRGK9F	XH-W1209	$5.00
Camera	PS3 Eye	https://ebay.us/8GwtOX	7010571	$15.00
Micromanipulator	3D printed (ABS)	https://reiserlab.github.io/Component-Designs/tether/micromanipulator		$20.00
Screw	M3x0.5 40mm	https://www.mcmaster.com/91287A028/	91287A028	$8.09
Locking nuts	M3x0.5	https://www.mcmaster.com/90576A102/	90576A102	$3.57
Nuts	M3x0.5	https://www.mcmaster.com/90592A085/	90592A085	$0.88
Washer	M3	https://www.mcmaster.com/95610A530/	95610A530	$3.81
Nuts	M6 to fix parts on Baseplate	https://www.mcmaster.com/90592A016/	90592A016	$2.71
Screws	M6	https://www.mcmaster.com/91274A138/	91274A138	$6.58
Washer	M6	https://www.mcmaster.com/91166A250/	91166A250	$3.17
Rubber feet	for Baseplate	https://www.amazon.com/dp/B06XPFDQBH		$11.00

Continued on next page

Table S2: *(continued)*

part	description	link	PN	price
Sphere holder	3D printed (ABS)	https://reiserlab.github.io/Component-Designs/walking/sphere-holder		$6.00
Tube fitting	air feed	https://www.mcmaster.com/51235K107/		$2.26
Tubing	1/4” OD	https://www.amazon.com/dp/B08JGVQY4S		$13.00
Sphere holder post	3D printed (ABS)	https://reiserlab.github.io/Component-Designs/walking/stands		$5.00
Lamp post and shade	3D printed (ABS)	https://reiserlab.github.io/Component-Designs/walking/illumination		$15.00
IR LED	940nm 5mm LED	https://www.digikey.com/short/5hnfw5bq	IR204	$0.90
Power supply	Any 5V power source	https://www.adafruit.com/product/276	276	$8.00
Tube Clamp	Keck Roller Clamp	https://www.usplastic.com/catalog/item.aspx?itemid=31260	16004	$5.00
Lens	25mm M12 Lens	http://www.m12lenses.com/25mm-F2-4-5MP-CCTV-Lens-p/pt-2524mp5y.htm	PT-2524MP5Y	$24.25
Lens holder	M12 Lens holder	http://www.m12lenses.com/M12-Lens-Holder-Plastic-p/pt-lh001p.htm	PT-LH001P	$3.00
Lens extension	M12 Macro Extension	http://www.m12lenses.com/7mm-Metal-Extension-Ring-for-M12-Mount-p/pt-erm12-700m.htm	PT-ERM12-700M	$3.00

Table S3: Examples for components used in typical preparatory setups.

part	description	link	PN	price
Fly picker wand	vacuum wand body	https://stores.netmotionstore.com/c001/	C001	$197.00
Fly picker tip	vacuum wand nozzle	https://stores.netmotionstore.com/2603/	2603	$28.00
Fly picker vacuum	vacuum pump	https://stores.netmotionstore.com/fv10110/	FV10110	$178.00
sarcophagus	custom machined from brass	https://reiserlab.github.io/Component-Designs/tether/sarcophagus#machined-brass-sarcophagus	JF-MR-FS0006/7	$600.00
cooling plate	TECA liquid cooled thermoelectric plate	https://www.thermoelectric.com/cold-plates/general-use-liquid-cooled/lhp-300cp-series/	LHP-300CP	$600.00
Temperature control	Benchtop temp. controller	https://www.ovenind.com/product/5r6-900/	5R6-900	$1135.00
water cooler	thermoelectic recirculating chiller	https://www.thermotekusa.com/product.php?pid=108	T257P	$3470.00
microscope	various; any dissecting scope			
breadboard base	Aluminum 10” x 12”	https://www.thorlabs.com/thorproduct.cfm?partnumber=MB1012	MB1012	$139.00
manipulator	4 axis manipulator	https://www.siskiyou.com/mx160l-14650000e.html	MX160L	$1500.00

Continued on next page

Table S3: *(continued)*

part	description	link	PN	price
tether adapter	connector (gold plated)	https://www.digikey.com/en/products/detail/te-connectivity-aerospace-defense-and-marine/205089-1/132229	A2160-ND	$32.20
tether	4 inch long, 0.005” diameter, tungsten rod	https://www.a-msystems.com/p-728-tungsten-rod.aspx	716100	$62.00
tether shaft	Hypodermic tubing	https://www.mcmaster.com/8988K65-8988K423/	304-TW 23 GA	$5.59
tether mount	brass rod + pin socket	https://www.digikey.com/en/products/detail/te-connectivity-aerospace-defense-and-marine/205090-1/132232?s=N4IgTCBcDaIIJgIwDZEFoByAREBdAvkA	A2161-ND	$1.11
glue	glass to glass adhesive	http://www.kemxert.com/product-catalog.cfm?pg=product-catalog&prd_pct_id=2	KOA-300	$30.00
UV curing light	UV curing system, 365 nm	https://www.thorlabs.com/thorproduct.cfm?partnumber=CS20K2	CS20K2	$2400.00
UV protective glasses	different manufacturers	https://amazon.com/dp/B01KJ9JKB6		$11.00
Funnel	3D printed (PLA, ABS)	https://reiserlab.github.io/Component-Designs/tether/funnels		$3.00
Paintbrush	fine tip	https://amazon.com/dp/B0878MN2VR		$7.00

Continued on next page

Table S3: *(continued)*

part	description	link	PN	price
Round bottom tube	Chilling tube	https://www.mcmaster.com/7012A54/	7012A54	$15.00
Hollow Body Pin Vise	Flyhook holder	https://www.mcmaster.com/8455A18/	8455A18	$16.00
Minutien Pin	Flyhook	https://amazon.com/dp/B00J5PMPJA		$17.00

Table S4: Examples for components used in typical experimental setups.

part	description	link	PN	price
Micromanipulator	miniature 4-axis micromanipulator, rotatable probe clamp	https://www.siskiyou.com/mx10-series-manipulator.html	MX10R	$575.00
tether mount	brass rod + pin socket	https://www.digikey.com/en/products/detail/te-connectivity-aerospace-defense-and-marine/205090-1/132232?s=N4IgTCBcDaIIJgIwDZEFoByAREBdAvkA	A2161-ND	$10.00
Sphere	milled or filed	https://www.generalplastics.com/products/fr-7100	FR-7120	
Sphere holder	custom machined			$400.00
mass flow controller	digital MFC	https://www.sierrainstruments.com/products/50series.html	SmartTrak 50	$1600.00
camera	Basler Ace USB3 camera, any with low res, hi FPS	https://www.baslerweb.com/en/products/cameras/area-scan-cameras/ace/#framerate=100;monocolor=mono;interface=usb30	Ace U acA640-750um	$402.00

Continued on next page

Table S4: *(continued)*

**part**	**description**	**link**	**PN**	**price**
lens	Computar Macro Zoom lens	https://computar.com/product/559/MLM3X-MP	MLM3X-MP	$625.00
illumination	2 LED plus a controller (FlyFizz suggestion)	https://wiki.janelia.org/wiki/display/flyfizz/Fly-on-a-ball#Fly-on-a-ball-Hardwareassembly		$385.00
temperature control	thermostat and small heater			$80.00
display 1	G3 system	https://reiserlab.github.io/Modular-LED-Display/Generation%203/		$2200.00
display 2	Projector options	https://www.ti.com/tool/DLPDLCR2010EVM	DLPDLC-R2010EVM	$499.00
breadboard base	Aluminum 10" x 12"	https://www.thorlabs.com/thorproduct.cfm?partnumber=MB1012	MB1012	$139.00
other thorlabs parts	rails, posts, rail cariers, etc.			$200.00
Rubber feet	for Baseplate	https://www.amazon.com/dp/B06XPFDQBH		$11.00

This document contains the supplements for 'Loesche, F., and Reiser, M.B. (2021). An Inexpensive, High-Precision, Modular Spherical Treadmill Setup Optimized for Drosophila Experiments. *Front. Behav. Neurosci.* 15:689573. doi: 10.3389/fnbeh.2021.689573'. It is shared under the same license Creative Commons Attribution License (CC BY). Refer to the main documents for details.
